# Bamboo‐Like Whiskers‐Reinforced Bioceramics Accelerate Large Segmental Bone Regeneration via Dual Modulation of Type‐H Vessels and Osteoinduction

**DOI:** 10.1002/advs.75178

**Published:** 2026-04-07

**Authors:** Cong Feng, Haibo Teng, Chuyao Xu, Jiaze He, Keting Liu, Xueying Li, Kai Zhang, Xiangdong Zhu, Xiangfeng Li, Jianguo Xu, Xingdong Zhang

**Affiliations:** ^1^ National Engineering Research Center for Biomaterials Department of Neurosurgery West China Hospital Sichuan University Chengdu China

**Keywords:** bamboo‐like whiskers, calcium phosphate ceramics, mechanical strength, segmental bone defect, type‐H vessels

## Abstract

Regenerative repair of segmental bone defect remains a major clinical challenge. The conventional mental implants suffer from mechanical strength mismatch and long‐term foreign bodies presence. While, the osteoinductive materials lack insufficient mechanical strength for the repair of load‐bearing bone. Inspired by bamboo, whisker‐reinforced Ca‐P ceramics (HW) have been developed to accelerate segmental bone regeneration. Bamboo‐like whiskers can reduce the stress concentration and impede crack propagation via whisker broken, whisker bridging, and crack deflection, achieving 3 times increase in the compressive strength compared to traditional ceramics. In addition, HW significantly promotes the formation of type‐H endothelial cells (ECs) via the integrin‐mediated HIF‐1 signaling pathway, which can secrete coupling factors (e.g. Noggin), to promote the osteogenic differentiation of bone marrow mesenchymal stem cells (BMSCs). In turn, HW can stimulate BMSCs to secrete coupling factors (e.g. VEGF) to support angiogenesis. In the segmental bone defect model, DFO is introduced to bamboo‐like whiskers (HW+DFO) to enhance type‐H vessels formation. HW+DFO can effectively promote the repair of segmental bone defects with mechanical recovery reaching 40% of that of normal femur. In conclusion, this study proposes a novel strategy for promoting bone regeneration by regulating type‐H vessels, offering a potential solution for addressing segmental bone defects.

## Graphical abstract

The graphical abstract image of bamboo‐like whisker‐reinforced Ca‐P bioceramics accelerating large segmental bone regeneration.

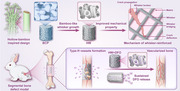

## 1 Introduction

The regenerative repair of segmental bone defects caused by acute trauma, infection, bone tumor excision, and congenital bone disease remains a problematic issue in the clinical field [1, 2]. Current clinical approaches for segmental bone defects mainly include vascularized bone grafting, distraction osteogenesis, membrane guided technique, and intramedullary nailing [3, 4]. Vascularized bone grafts preserve blood supply to promote bone healing, while the disadvantages include limited availability of grafts, insufficient integration into the damaged bone, donor site morbidity and so on [5]. Distraction osteogenesis and membrane guided technique have demonstrated reliable efficacy in the repair of segmental bone defect, but the multiple surgical interventions, patient discomfort, the prolonged treatment periods, and the risks of bone nonunion or infection have restricted the applications [6]. Intramedullary nailing system, including rods, screws, and plates, is widely used in clinical practice to stabilize and align the bone [7], but their bioinert nature results in poor osseointegration. Although the surface modification strategies can impart bioactivity to metallic implants [8], these implants remain non‐biodegradable and are associated with risks of stress shielding and long‐term loosening failure Therefore, how the prosthesis promotes the regenerative repair of segmental bone defects remains an unsolved problem. To the best of our knowledge, the essential requirements for ideal implants to repair segmental bone defects are as follows: 1) appropriate mechanical properties to provide initial mechanical support; 2) appropriate biodegradability; and 3) exceptional osteogenesis and angiogenesis.

Tissue‐inducing biomaterials designed to induce the regeneration of damaged or missing tissues or organs without the addition of cells and/or bioactive factors, which have been list into the definition of biomaterials for the twenty‐first century [9]. Osteoinductive calcium phosphate (Ca‐P) ceramic, as a representative tissue‐inducing biomaterial, have been clinically applied in China for many years with favorable outcomes [10, 11]. However, their clinical utility is primarily limited to repairing bone defects in non‐load‐bearing sites. The inherent brittleness and low mechanical properties of porous Ca‐P ceramics may lead to risks of collapse and fracture during the repair of segment bone defects in load‐bearing sites [12, 13]. Generally, the synergistic enhancement of mechanical properties and bioactivity represents a contradictory in Ca‐P bioceramics. For instance, the high sintering temperature can improve mechanical properties at the expense of bioactivity of the bioceramics. Second‐phase whisker reinforcement is a common strategy to improve the mechanical property of ceramics. Previous studies have demonstrated that the incorporation of second phase such as carbon nanotubes, alumina, calcium oxide, and bioinert whiskers into the ceramic matrix could improve the mechanic strength of ceramics [14, 15, 16]. Unfortunately, the inhomogeneous distribution and the whisker agglomeration resulted in limited mechanical enhancement, and the incorporation of the inert phase usually reduces the bioactivity of ceramics.

In situ whisker reinforcement mainly refers to the growth of whiskers within ceramic matrix with the addition of the specific whisker growth agents through the hydrothermal treatment. Our group previously developed in situ formation of hydroxyapatite (HA) whiskers within Ca‐P ceramics, achieving uniform whisker dispersion in the ceramic matrix and significantly enhancing their mechanical properties [17]. During the hydrothermal treatment, Ca‐P ceramics can release Ca^2+^ and PO_4_^3−^ while simultaneously promoting the nucleation of HA crystals on surface. Generally, HA crystals preferentially attach the *c* crystal surface (negative potential) to *a* and *b* positive surfaces, and rapidly grow along the *c* axis, resulting the HA whiskers [18, 19]. However, the crystal structure of HA whiskers is almost intact, lacking crystalline defects such as grain boundaries and dislocations, which results in relatively low bioactivity [20]. Therefore, improving the bioactivity of in situ whisker while maintain its mechanical strength remains challenging. Bamboo is known for its unique hollow tubular architecture, which may play a crucial role in its growth and is generally associated with notable toughness. Under strong wind conditions, bamboo undergoes flexural deformation to dissipate stress, effectively resisting breakage. Such stress redistribution through the hollow tubular design facilitates environmental adaptation, offering innovative concepts for biomimetic bone repair scaffold development [21, 22]. Moreover, hollow tubes facilitate nutrient transport and may exhibit satisfactory bioactivity compared to the HA solid whiskers.

During the regenerative repair of segmental bone defects, the rapid angiogenesis is crucial. It is noteworthy that segmental bone defects are accompanied by vascular disruption, leading to acute necrosis, hypoxia, and hematomas [23]. In recent years, two types of blood vessels are distinguished in the bone: type‐H vessels and type‐L vessels. Type‐H vessels are characterized by high expression of CD31 and endothelial mucin (EMCN). In contrast, type‐L vessels express low levels of CD31 and EMCN [24]. Oxygen rich blood flows from arteries and directly into type‐H vessels. Blood then continues into type‐L vessels, ultimately draining into the central vein. It is confirmed that the majority of Osterix^+^ osteoprogenitor cells, Runx2^+^ early osteoprogenitors, and collagen type 1α^+^ osteoblast cells densely localize around type‐H vessels, not type‐L vessels [25]. Moreover, type‐H vessels can secrete various factors, including transforming growth factor β (TGF‐β), platelet‐derived growth factor (PDGF), fibroblast growth factor 1 (FGF‐1), Noggin, and vascular endothelial growth factor (VEGF), to stimulate the proliferation and differentiation of osteoprogenitor cells [24, 25, 26]. Notably, during the early stage of bone injury, type‐H vessels are extensively distributed around the growing trabecular bones near the frontier growth area, indicating type‐H vessels promote new bone formation [27]. Numerous researches have targeted type‐H vessels to enhance osteogenesis‐angiogenic coupling, thereby accelerating the repair of segmental bone defects [28]. Zeng et al. [29] have confirmed that the sustained release of deferoxamine (DFO) can promote the formation of type‐H vessels, thereby accelerating the regenerative repair of segmental bone defects. Our previous studies have demonstrated that type‐H vessels play a crucial role in the osteoinduction of Ca‐P ceramics, and biphasic calcium phosphate (BCP) ceramics can significantly promote the formation of type‐H endothelial cells (type‐H ECs) via upregulating integrin‐related expression and subsequently activating downstream signaling pathways, such as PI3K‐Akt, and cGMP‐PKG [30]. Therefore, the development of Ca‐P ceramics with dual modulation of rapid type‐H vascularization and osteoinduction holds great promise for the regenerative repair of segmental bone defect.

**SCHEME 1:**
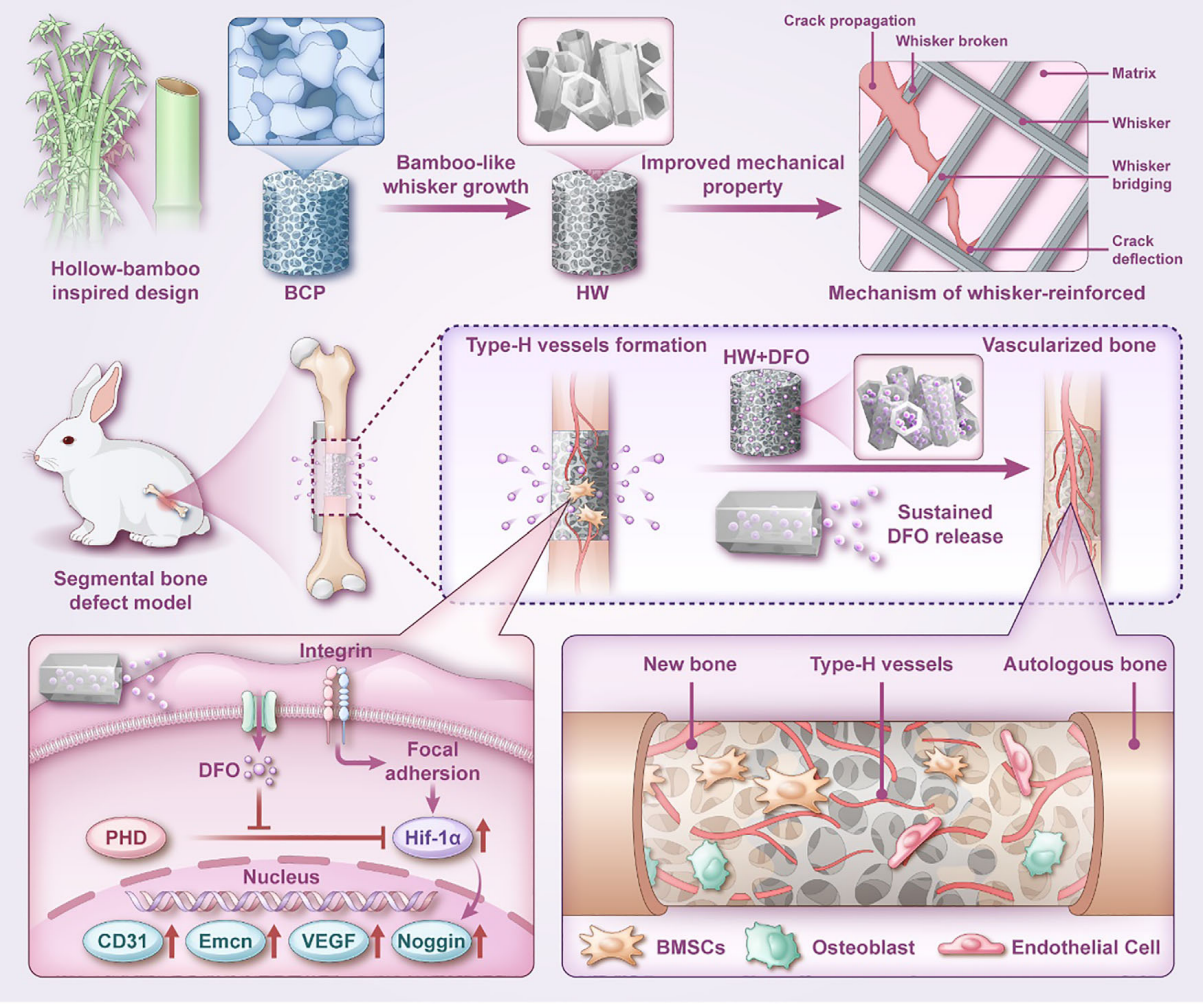
The sketch map of bamboo‐like whisker‐reinforced Ca‐P bioceramics accelerating large segmental bone regeneration.

Inspired by bamboo, the in situ bamboo‐like whisker structure was first constructed via hydrothermal treatment to simultaneously enhance the mechanical properties and osteoinduction of Ca‐P ceramics (Scheme 1). In vitro co‐culture model using mouse bone marrow mesenchymal stem cells (BMSCs) and rat endothelial progenitor cells (EPCs) was established to elucidate the biological mechanisms underlying the coupling of osteogenesis and angiogenesis. In vivo osteoinduction and type‐H vessels formation were evaluated using a mice intramuscular implantation model. Finally, a large segmental bone defect model in rabbits was constructed to evaluate the repair efficacy of the novel functionalized bioceramic scaffolds in load‐bearing sites. Furthermore, DFO was loaded into the in situ bamboo‐like whiskers utilizing capillary force to highlights the significance of type‐H vessels formation in the large segmental bone regeneration.

## 2 Results

### 2.1 Material Characterization

**FIGURE 1:**
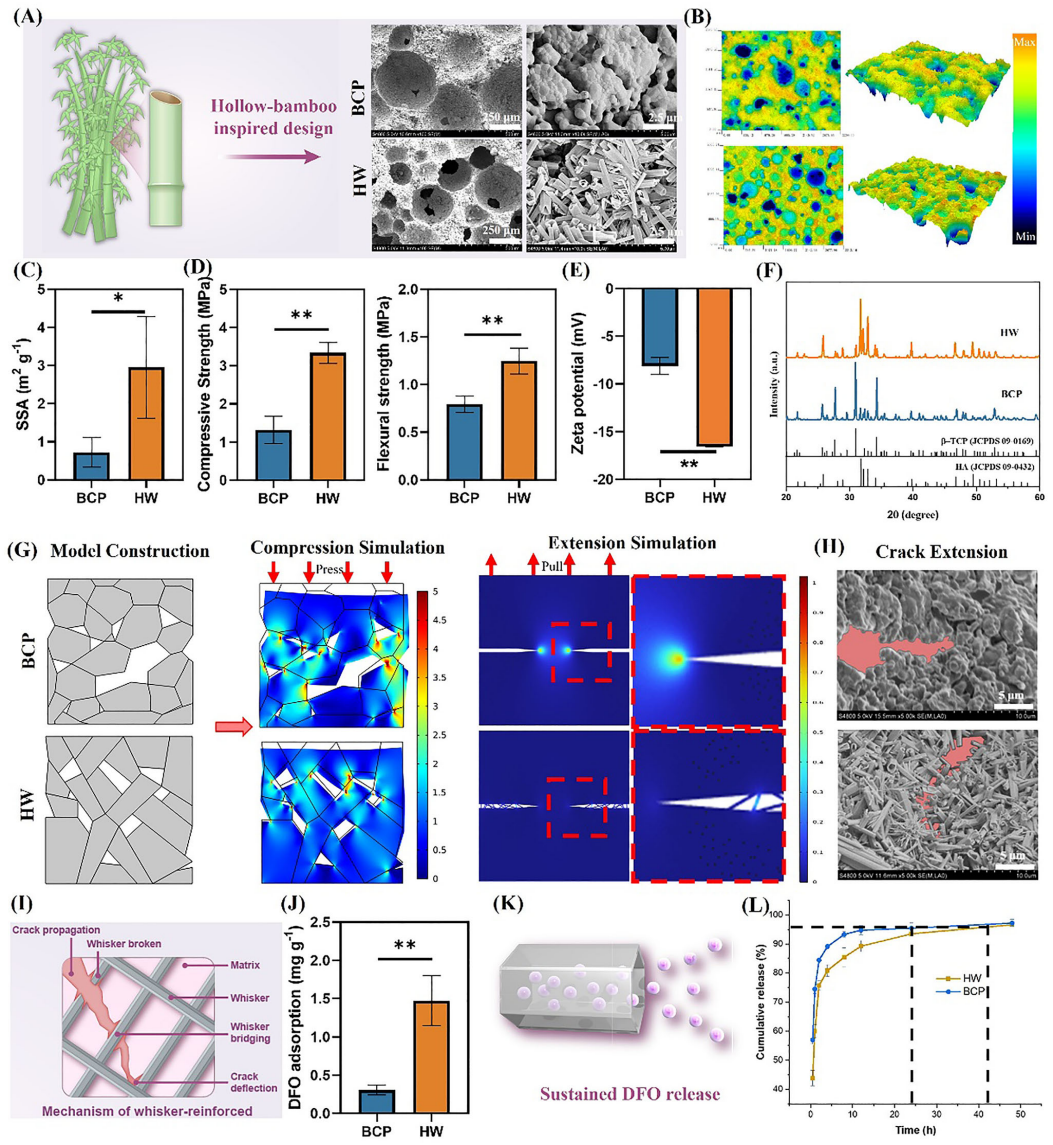
(A) Schematic diagram of bamboo inspired design and the representative SEM images of BCP and HW. (B) Surface roughness, (C) specific surface area, (D) compressive strength and flexural strength, (E) Zeta potential, and (F) XRD patterns of BCP and HW (n = 3). (G) Finite element analysis (FEA) model, compression simulation, and extension simulation of BCP and HW. (H) SEM images of crack extension (red, crack). (I) Mechanism diagram of in situ bamboo‐like whisker‐reinforced mechanical properties of Ca‐P bioceramics. (J) DFO adsorption BCP and HW (n = 3). (K) Schematic diagram of the sustained release assay of HW. (L) DFO releasing of BCP and HW (n = 3). Values are expressed as the mean ± SD. The unpaired two‐tailed Student's *t*‐test was used in (C), (D), (E), and (J). ^*^ *p* < 0.05 and ^**^ *p* < 0.01.

The bamboo‐like whisker‐modified BCP ceramics (HW) were originally inspired by bamboo (Figure 1A). SEM images revealed that both BCP and HW ceramics exhibited the interconnected porous structures. After hydrothermal treatment, in situ bamboo‐like whiskers (length: 2.33 ± 0.57 µm, diameter: 0.59 ± 0.19 µm, aspect ratio: 3.30 ± 0.91) were successfully constructed on HW. It was worth noting that the in situ bamboo‐like whiskers were distributed throughout the ceramics (Figure S1). 3D profilometer confirmed that the surface roughness of HW was higher than that of BCP (Figure 1B). As exhibited in Figure 1C, HW possessed a higher specific surface area than BCP. The cumulative release curves demonstrated that the Ca^2^^+^ concentration of HW was lower compared to BCP, indicating that HW may promote the formation of bone‐like apatite (Figure S2). Furthermore, HW exhibited a significantly lower zeta potential compared to BCP (Figure 1E). XRD patterns indicated that both BCP and HW were composed of HA and β‐TCP phases. The HA phase ratio (81%) in HW was higher than in BCP (17%) by calculation (Figure 1F). Moreover, HW had the relatively higher DFO adsorbing ability than BCP (Figure 1J). The construction of the in situ bamboo‐like whiskers could significantly increase the DFO releasing ability of BCP ceramics (Figure 1K), and the releasing duration of HW was nearly double times higher than BCP (Figure 1L).

To investigate the effect of in situ bamboo‐like whiskers on the mechanical properties of ceramics, compression tests and three‐point bending tests were conducted on BCP and HW. As shown in Figure 1D and Figure S3, the compressive strength of BCP was 1.32 ± 0.36 MPa, while that of HW increased by ≈ 3 times to 3.34 ± 0.28 MPa. Moreover, the elastic modulus of HW was significantly higher than that of BCP. HW also showed the significant superior flexural strength compared to BCP. These results indicated that the construction of in situ bamboo‐like whiskers could significantly enhance the mechanical properties of BCP ceramics. To further understand the mechanism of in situ whisker reinforcement, a 2D model was designed. The calculated Mises stress for the whisker‐free structure was 24.30 MPa, while the Mises stress for the whisker‐reinforced structure decreased to 14.20 MPa. Furthermore, the presence of in situ whiskers significantly reduced stress concentration. A crack propagation model was established to simulate material failure under tensile loading. As shown in Figure 1G, the crack initiation occurred at 50 MPa in the without whisker structure, whereas crack propagation began at 69 MPa in the in situ whisker‐reinforced scaffold. Based on the finite element analysis (FEA) results, in situ bamboo‐like whiskers could effectively mitigate stress concentration and imped crack propagation, thereby enhancing the mechanical properties of the ceramics. Fracture morphology indicated that in situ bamboo‐like whiskers could promote crack deflection via whisker bridging and whisker broken (Figure 1H,I).

### 2.2 In Vitro Cellular Responses of EPCs

**FIGURE 2:**
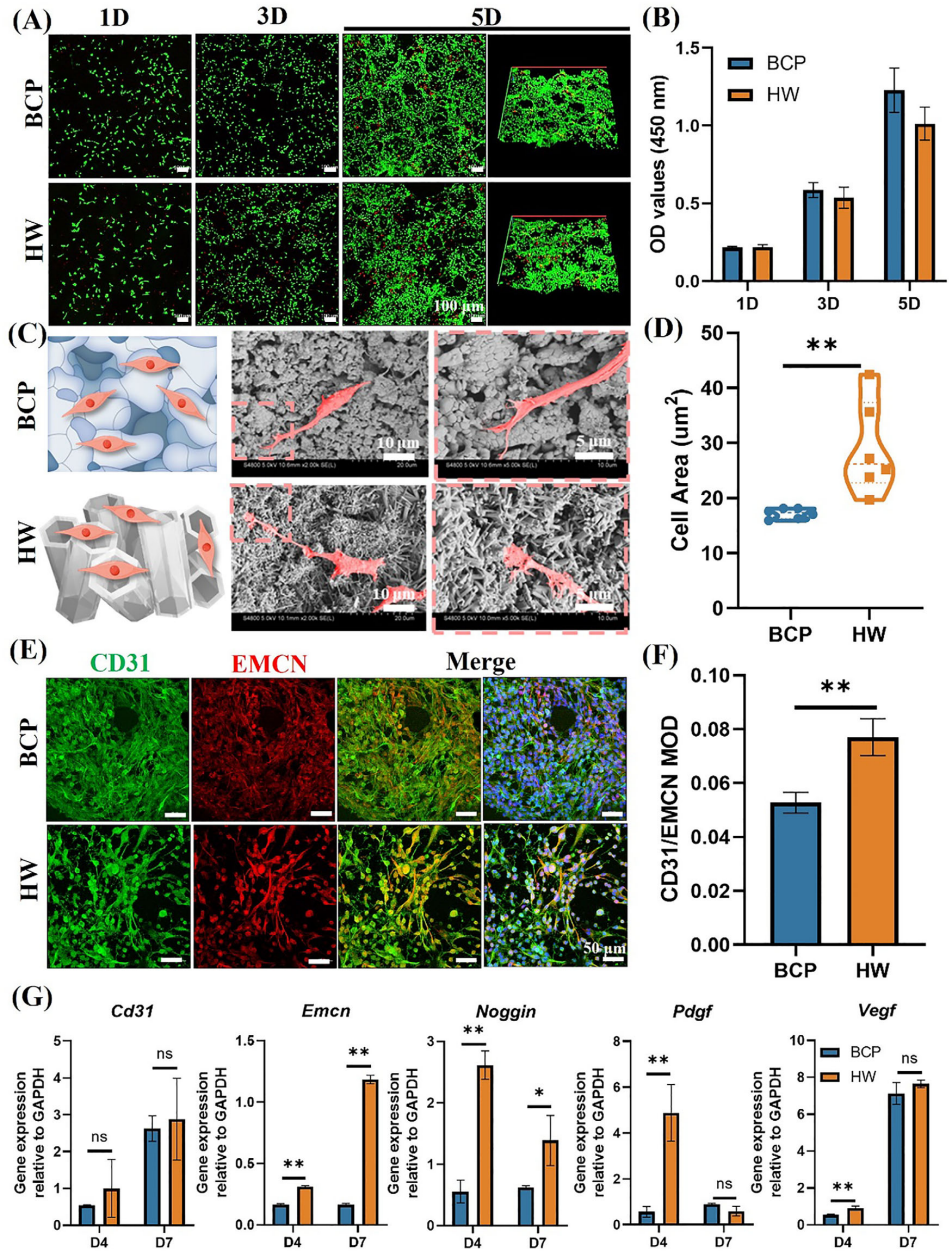
(A) CLSM images, and (B) CCK‐8 results of EPCs culturing on BCP and HW at 1, 3, and 5 days (n = 5). (C) Schematic representation and SEM images for the morphology of EPCs seeded on the BCP and HW for 2 days. (D) Cell area measurement of EPCs (n = 6). (E) Immunofluorescent staining of CD31 (red), EMCN (green), and nuclei (blue) after EPCs seeded on the BCP and HW for 7 days. (F) MOD values of CD31/EMCN (n = 3). (G) The relative gene expression (e.g., *Cd31, Emcn, Noggin*, and *Vegf*) of EPCs on the BCP and HW for 4, and 7 days (n = 3). Values are expressed as the mean ± SD. The unpaired two‐tailed Student's *t*‐test was used in (B), (D), (F), and (G). ^*^ *p* < 0.05 and ^**^ *p* < 0.01.

CLSM observations (Figure 2A) reveals that both BCP and HW could promote the adhesion and proliferation of EPCs on their surfaces. EPCs had nearly covered the entire surface and invaded the internal pores of the ceramics at day 5. CCK8 results (Figure 2B) further indicated that BCP and HW enhanced the proliferation of EPCs. SEM images (Figure 2C) reveals that EPCs extended abundant pseudopodia to grasp ceramic grains or in situ bamboo‐like whiskers after 2 days of culture. By quantitative analysis, HW could significantly promote the spreading of EPCs compared with BCP (Figure 2D). The differentiation of EPCs after co‐culture with the materials was evaluated by immunofluorescence staining of type‐H vessel (CD31 and EMCN). After 7 days of culture, both BCP and HW promoted the expression of CD31 and ECMN (Figure 2E). Quantitative analysis revealed HW significantly enhanced the expression of CD31 and ECMN compared with BCP (Figure 2F). Moreover, HW exhibited significantly higher expression of *Emcn*, *Vegf*, *Pdgf*, and *Noggin* than BCP after culturing for 4 days. HW also significantly promoted the expression of *Emcn* and *Noggin* compared to BCP with prolonging the culture time to day 7 (Figure 2G). In addition, the expression of *Cd31* was slightly higher in the HW group than in the BCP group, but the difference was not statistically significant. qRT‐PCR results were consistent with the immunofluorescence staining findings. These results demonstrated that HW with in situ bamboo‐like whisker structures could promote the differentiation of EPCs into type‐H endothelial cells.

### 2.3 The Osteo‐Angiogenic Coupling Effect of EPCs and BMSCs

**FIGURE 3:**
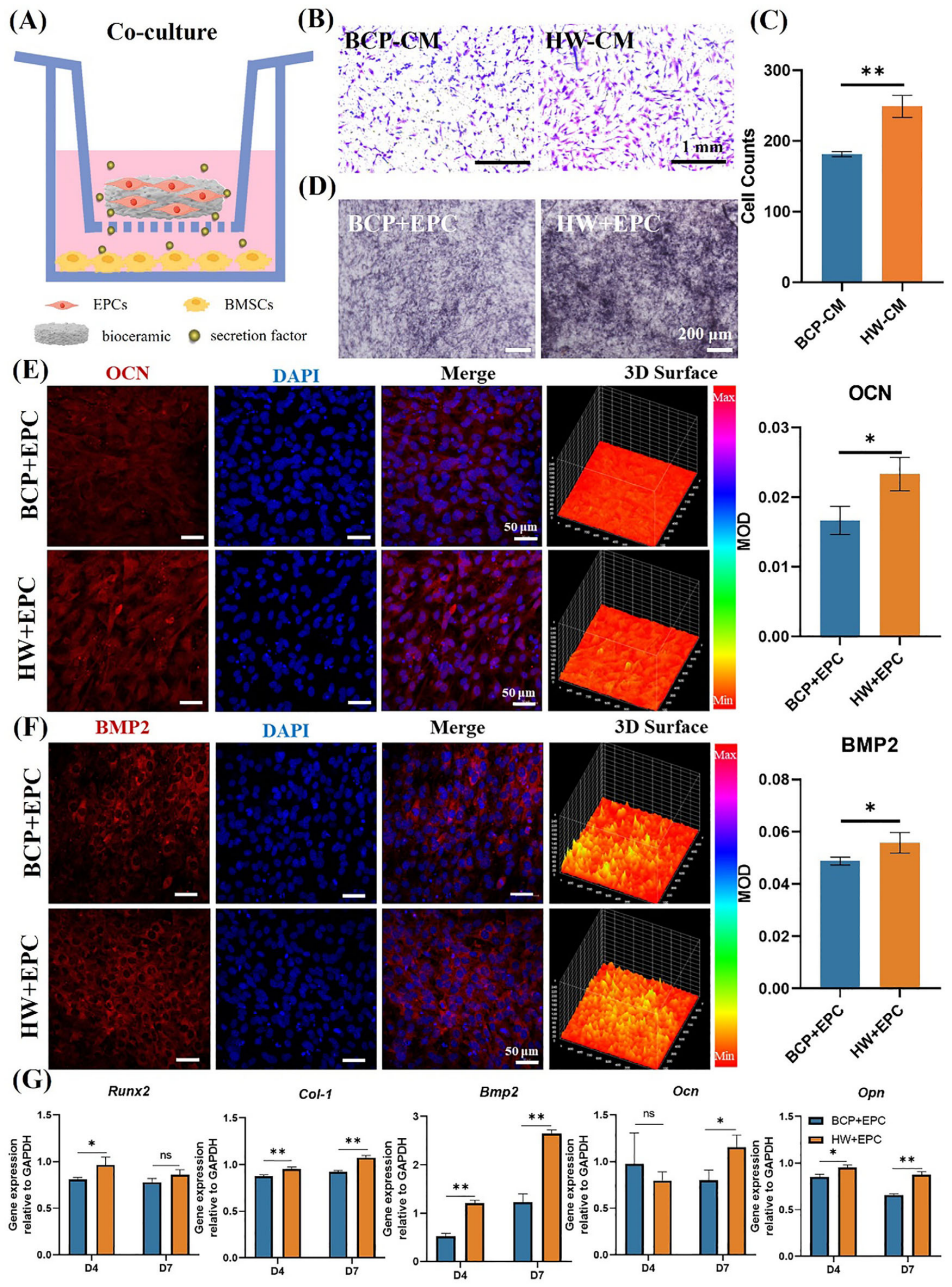
(A) Schematic diagram of bioceramics facilitating the crosstalk of EPCs and BMSCs. (B) Transwell assay showing BMSCs migration capacity with crystal violet staining after cultured in different CMs. (C) Quantitative analysis of number of segments (n = 3). (D) ALP staining analysis of BMSCs in the co‐culture system after 4 days. Immunofluorescent staining and MOD values of OCN (E) and BMP2 in the co‐culture system (F) (n = 3). (G) Osteogenic gene expression (e.g., *Runx2, Col‐1, Bmp2, Ocn*, and *Opn*) of BMSCs in the co‐culture system (n = 3). Values are expressed as the mean ± SD. The unpaired two‐tailed Student's *t*‐test was used in (C), (E), (F), and (G). ^*^ *p* < 0.05, ^**^ *p* < 0.01.

To investigate the osteo‐angiogenic coupling effect in vitro, a co‐culture system that placed ceramics and EPCs in the upper chamber and seeded BMSCs in the lower chamber (Figure 3A) was employed to explore the effect of material‐mediated differentiation of EPCs on BMSCs. First, the Trans‐well assay was employed to investigate the effect of different conditioned mediums (CMs) on the migration of BMSC. The results (Figure 3B,C) demonstrates that HW‐CM significantly enhanced the migration capacity of BMSCs. After co‐culturing for 4 days, the HW+EPC co‐culture system significantly enhanced the expression of ALP protein in BMSCs compared to the BCP+EPC co‐culture system (Figure 3D). Notably, the BCP and HW extract groups exhibited significantly weaker ALP expression than the HW+EPC and BCP+EPC groups from the ALP staining (Figure S4). The expression of osteogenic markers (i.e. OCN and BMP2) was also detected by immunofluorescence staining to evaluate the osteogenic differentiation of BMSCs (Figure 3E,F). Quantitative analysis revealed that HW+EPC significantly promoted high‐level productions of OCN and BMP2 compared to BCP+EPC. Moreover, HW+EPC significantly up‐regulated the expression of *Runx2*, *Col‐1*, *Bmp2*, and *Opn* genes on day 4 and increased the expression of *Col‐1*, *Bmp2*, *Ocn*, and *Opn* genes on day 7 compared with BCP+EPC from the qRT‐PCR results (Figure 3G). These results indicated that HW could mediate the differentiation of EPCs to secrete the related cytokines, which can crosstalk with BMSCs to promote the osteogenic differentiation of BMSCs.

**FIGURE 4:**
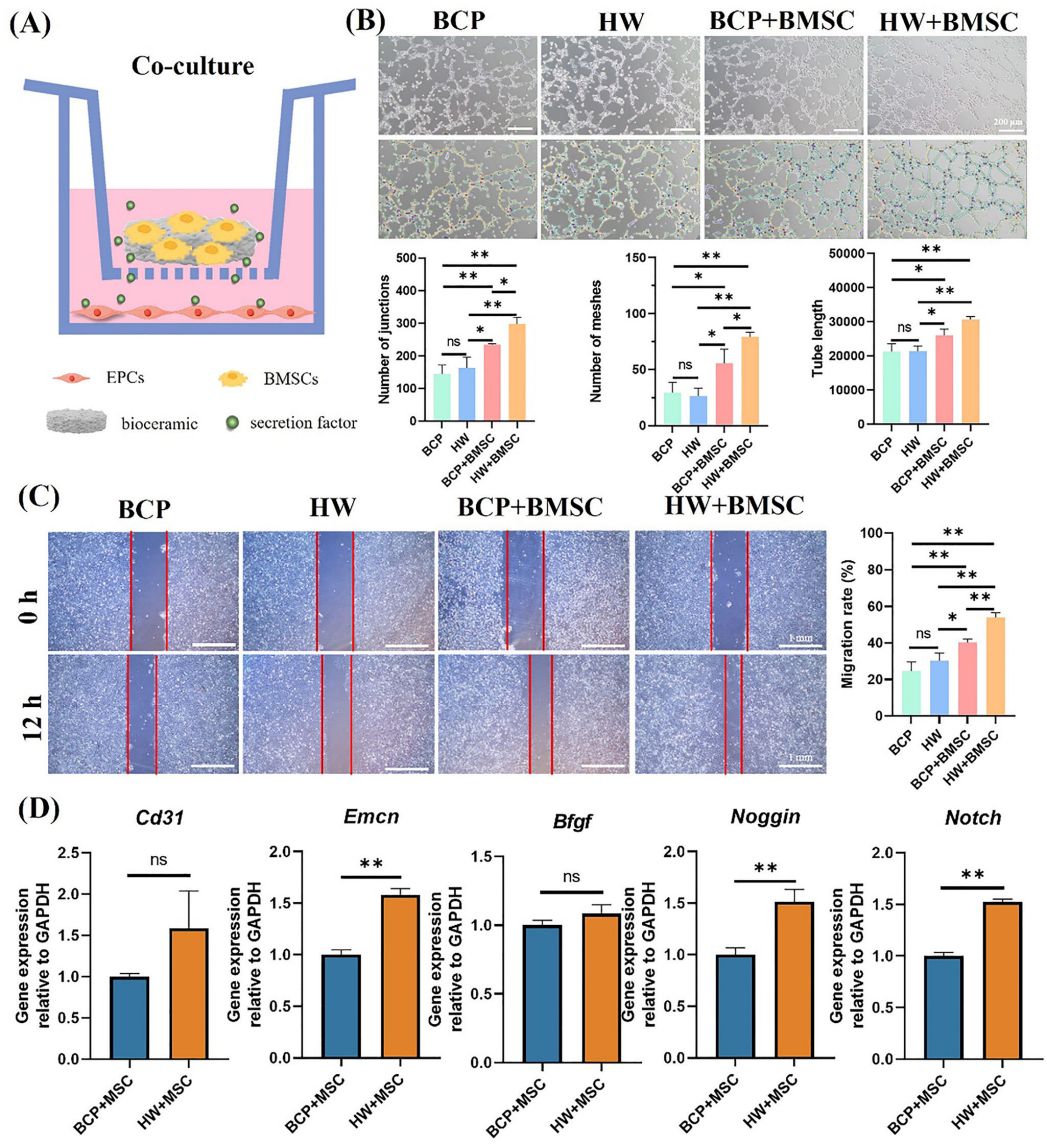
(A) Schematic diagram of bioceramics facilitating the crosstalk of BMSCs and EPCs. (B) Tube formation assay of EPCs, quantifying number of junctions, number of meshes, and tube length (n = 3). (C) Representative images from cell scratch assays performed on EPCs across various groups and quantification of cell migration through scratch (n = 3). (D) Angiogenic gene expression (e.g., *Cd31, Emcn, Bfgf, Noggin*, and *Notch*) of EPCs on 4 days in the co‐culture system (n = 3). Values are expressed as the mean ± SD. One‐way ANOVA with Tukey's post–hoc test was used for multiple comparisons in (B), and (C). The unpaired two‐tailed Student's *t*‐test was used in (D). ^*^ *p* < 0.05 and ^**^ *p* < 0.01.

Furthermore, a co‐culture system (Figure 4A) that placed ceramics and BMSCs in the upper chamber and seeded EPCs in the lower chamber was also employed to explore the effect of material‐mediated osteogenic differentiation of BMSCs on EPCs. The tube formation assay revealed that BCP+BMSC and HW+BMSC exhibited higher numbers and lengths of tubes compared to BCP and HW. Notably, the number and length of tubes in the HW+BMSC group were significantly greater than those in the other groups (Figure 4B). Moreover, the scratch assay indicated that the gap was significantly reduced in the BCP+BMSC and HW+BMSC groups compared to the BCP and HW groups after 12 h post‐scratch (Figure 4C). Quantitative migration analysis demonstrated that BCP+BMSC and HW+BMSC significantly increased the number of migrating cells compared to BCP and HW (Figure 4C). Additionally, the migration rate in HW+BMSC was significantly higher than that in the other three groups, suggesting that HW+BMSC enhanced the migratory ability of EPCs (Figure 4C). qRT‐PCR results showed that HW+BMSC could significantly promote the expression of angiogenic genes including *Emcn*, *Noggin*, and *Notch* compared with BCP+BMSC. No significant differences were observed in *Cd31* and *Bfgf* expression between the two groups (Figure 4D). These results indicated that HW could mediate osteogenic differentiation of BMSCs to secrete the related growth factors, thereby promoting tube formation and differentiation of EPCs. Moreover, the ELISA results showed that the key paracrine factors (such as Noggin, and VEGF) between EPCs and BMSCs in the HW group were significantly higher than that in the BCP group (Figure S5).

### 2.4 Transcriptome Sequencing Analysis

**FIGURE 5:**
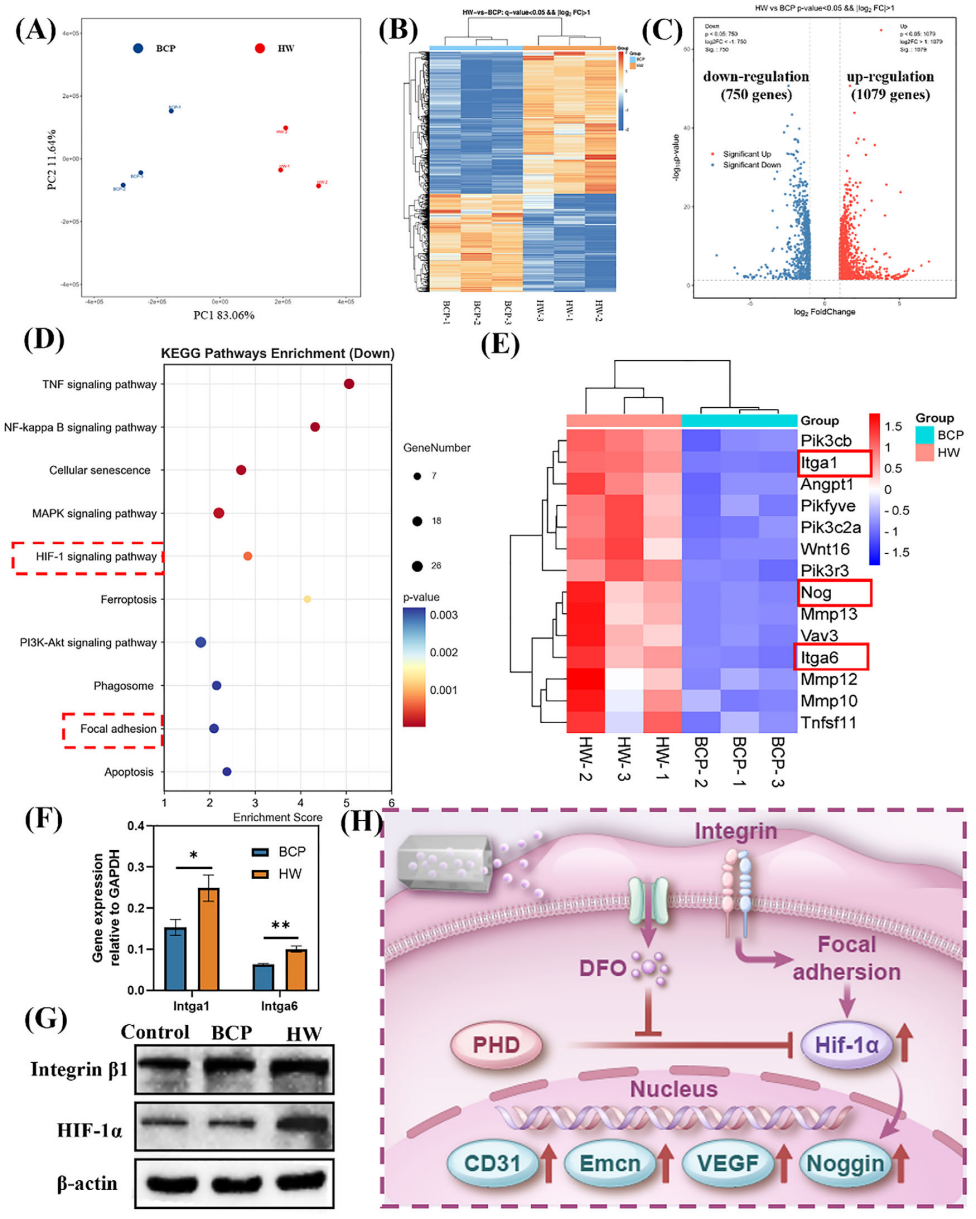
RNA sequencing analysis of EPCs cultured on BCP and HW. (A) PCA analysis of BCP and HW groups. (B) Heatmap of differentially expressed genes of BCP and HW groups. (C) Volcano plot of differentially expressed genes. (D) KEGG analysis. (E) Heatmap of differentially expressed genes related to cell adhesion and differentiation in transcriptome analysis. (F) qRT‐PCR analysis for integrin binding gene expressions of EPCs from different groups on day 4 (n = 3). (G) Western blotting of Integrin/HIF‐1α pathway related markers. (H) Mechanism illustration of EPC differentiation. Values are expressed as the mean ± SD. The unpaired two‐tailed Student's *t*‐test was used in (F). ^*^ *p* < 0.05, ^**^ *p* < 0.01.

RNA sequencing analysis was performed to elucidate the potential molecular mechanisms of EPC differentiation modulated by HW scaffolds. Principal component analysis (PCA) revealed high variability among sample groups, meeting the requirements for further analysis (Figure 5A). The gene expression heatmap demonstrated good parallelism within sample groups (Figure 5B). As shown in Figure 5C, HW group exhibited 1079 upregulated genes and 750 downregulated genes compared to BCP group. To further explore the underlying signaling pathways, KEGG enrichment analysis was conducted. Compared to BCP, HW showed enrichment in Focal adhesion and HIF‐1 signaling pathway (Figure 5D). Based on enrichment results, the expression of genes associated with Focal adhesion and HIF‐1 signaling pathway was subsequently focused on. As shown in Figure 5E, HW significantly upregulated the expression of cell adhesion‐related genes, such as *Itgα1* and *Itgα6*, compared to BCP. Moreover, qRT‐PCR confirmed the RNA sequencing results, demonstrating that HW significantly upregulated the expression of *Intgα5*, *Intgαv*, and *Intgb1* compared to BCP (Figure 5F; Figure S6). Western blot (WB) confirmed the upregulation of Integrinβ1 and HIF‐1α in the HW group compared with the BCP group (Figure 5G). Collectively, these findings indicated that the bamboo‐like whiskers could significantly enhance the adhesion and spreading of EPCs, and promote their differentiation into type‐H endothelial cells by upregulating the integrin‐mediated HIF‐1 signaling pathway (Figure 5H).

### 2.5 In Vivo Osteoinductivity in Mice Intramuscular Implantation

**FIGURE 6:**
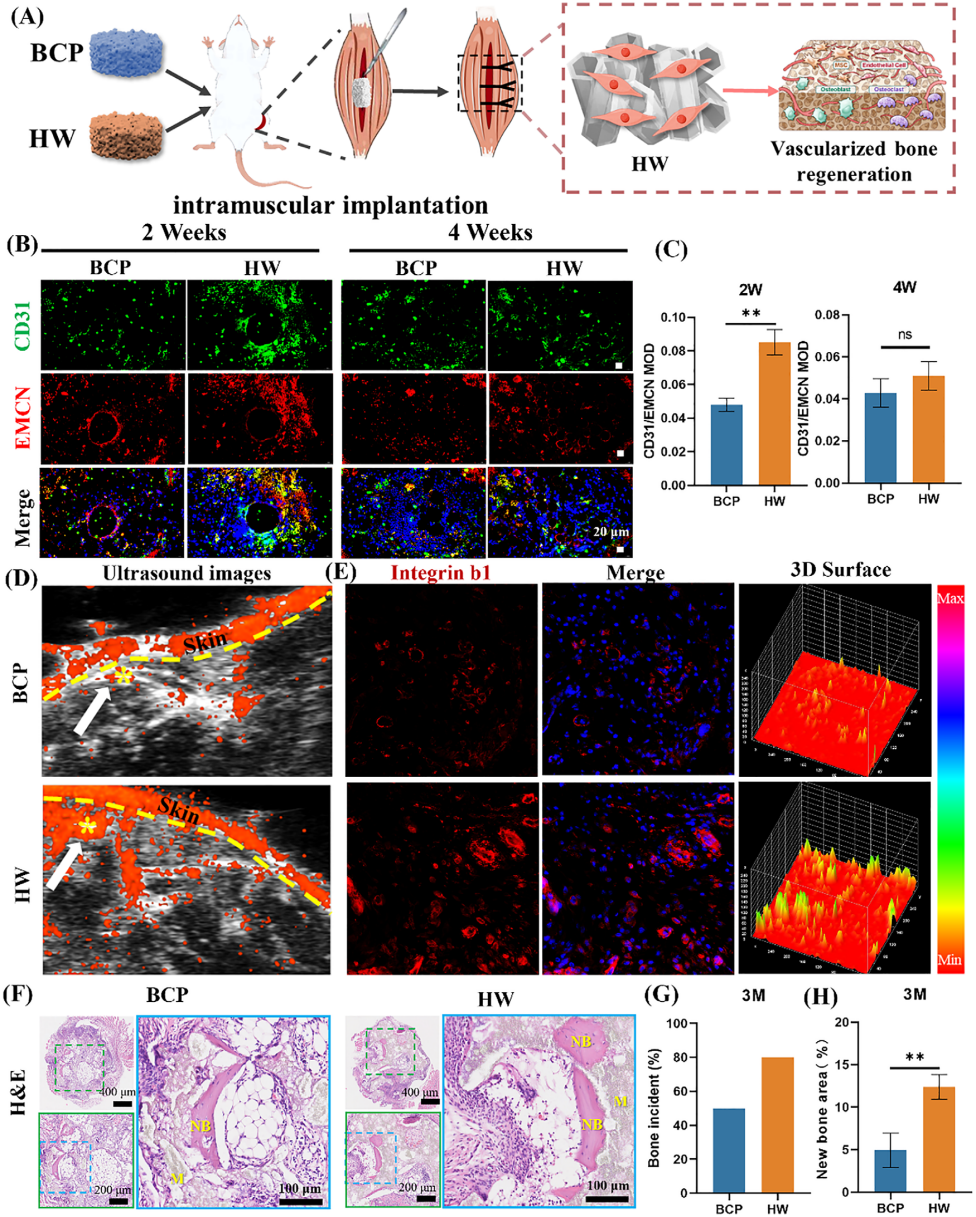
(A) Schematic diagram of BCP and HW implantation into the thigh muscular pouches of BALB/C mice. (B) CD31 and EMCN immunofluorescence staining of samples after implanting for 2 and 4 weeks, and (C) MOD values (n = 3). (D) After 14 days of implantation, ultrasound images of implanted bioceramics were obtained (Black asterisks and white arrowheads denote the bioceramics and vascular systems). (E) Immunofluorescence staining of Integrin b1 of samples after 2 weeks of implantation. (F) H&E staining of implanted samples after implanting for 3 months (M refers to material, and NB refers to new bone). Quantitative comparisons of (G) Bone incident, and (H) new bone area after implanting for 3 months (n = 6). Values are expressed as the mean ± SD. The unpaired two‐tailed Student's *t*‐test was used in (C), and (H). ^*^ *p* < 0.05 and ^**^ *p* < 0.01.

BCP and HW scaffolds were implanted into the thigh muscles of mice to evaluate their type‐H vessels formation and osteoinduction (Figure 6A). Immunofluorescence staining (Figure 6B) reveals that the expression level of type‐H vessels (labeled by CD31 and EMCN) in HW was significantly higher than that in BCP, indicating that HW could promote type‐H vessel formation after implanting for 2 weeks. However, the expression levels of CD31 and EMCN decreased after 4 weeks of implantation, suggesting that type‐H vessels inside the implant were remodeling into mature vessels (Figure 6C). Moreover, the ultrasound imaging was employed to assess the blood perfusion within the scaffolds. For HW group, robust blood flow signals were observed, indicating adequate blood supply inside the implant. In contrast, only minimal blood flow signals were detected in the BCP group (Figure 6D). Further histological analysis with H&E staining revealed the apparent ectopic bone formation in BCP and HW after implanting for 3 months., accompanied by abundant blood vessels. Additionally, the new bone formed in BCP was relatively independent, whereas the new bone formed in HW was interconnected, forming large continuous bone regions (Figure 6F). Quantitative analysis demonstrated that the new bone area and bone incident in HW were significantly higher than those in BCP after implanting for 3 months (Figure 6G,H). Moreover, HW could significantly raise the Integrin b1 and HIF‐1α expression at the determining time point of week 2 compared to BCP (Figure 6E; Figure S7), which was consistent with in vitro experiments. Immunofluorescent staining also showed that osterix^+^ osteoprogenitor cells were predominantly distributed around type‐H vessels (Figure S8).

### 2.6 In Vivo Bone Regeneration in Rabbit Critical‐Size Segmental Diaphyseal Defect

**FIGURE 7:**
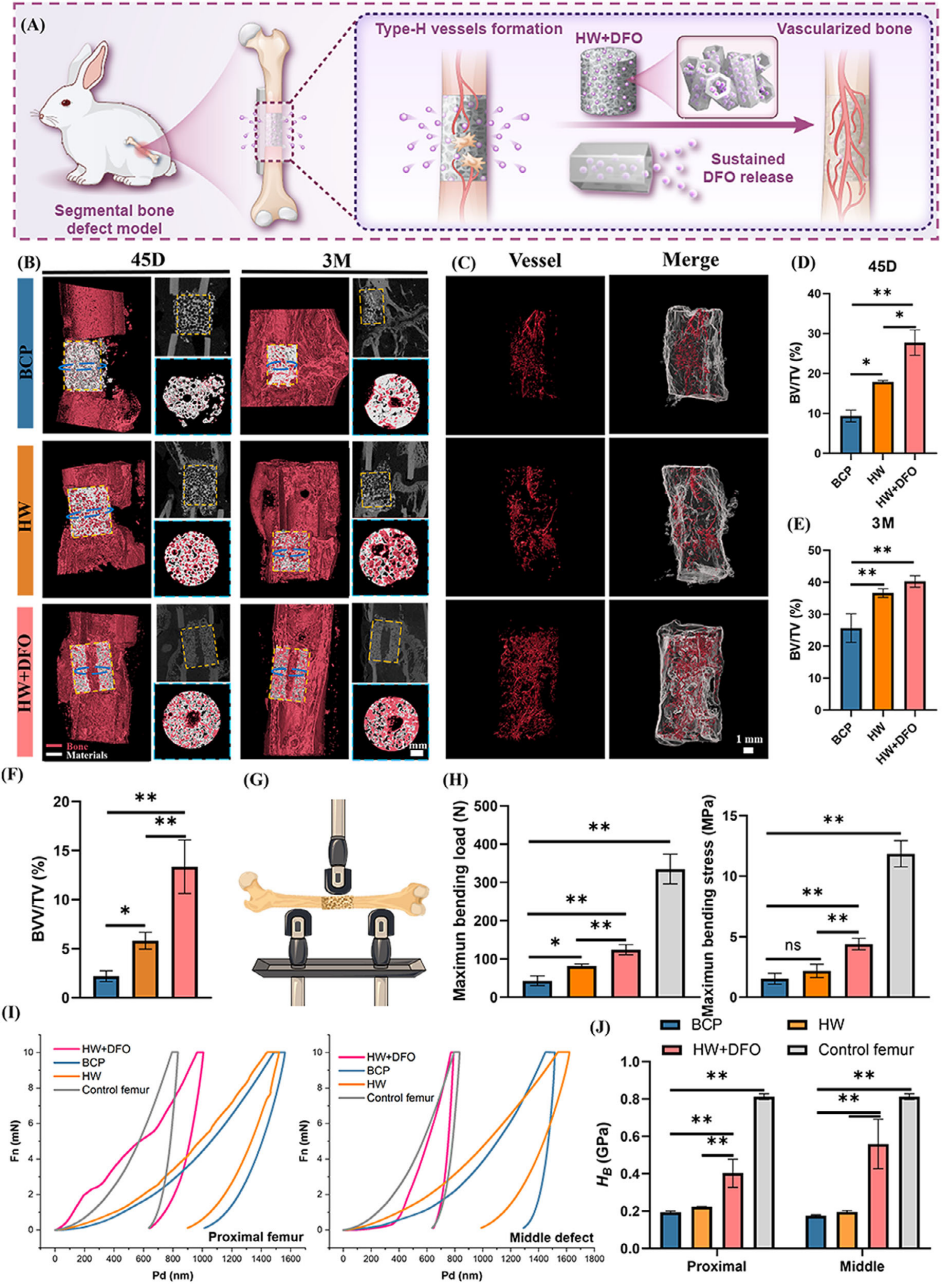
(A) Schematic diagram of the critical‐sized segmental bone defect model in rabbit femur. (B) Micro‐CT‐rendered 3D images and representative tomographic images (red, new bone; white, the remaining materials). (C) 3D reconstruction of new vessel formation in different groups. Quantitative comparisons of the bone volume fraction (BV/TV) after implanting for 45 days (D) and 3 months (E) in each group (n = 3). (F) Quantitative analysis of vessel regeneration‐related index (BVV/TV) after implanting for 3 months in each group (n = 3). (G) Schematic diagram of the three‐point bending. (H) Quantitative comparisons of the maximum bending load and the maximum bending stress. (I) Typical load‐displacement curves generated from nanoindentation tests on ingrown new bone. (J) Quantitative analysis of averaged hardness of new bone (n = 3). Values are expressed as the mean ± SD. One‐way ANOVA with Tukey's post hoc test was used for multiple comparisons in (D), (E), (F), (H), and (J). ^*^ *p* < 0.05 and ^**^ *p* < 0.01.

A critical‐sized segmental bone defect model of rabbit femur was developed (Figure 7A). Comprehensive insights into the regeneration of new bone tissue within the implant and the healing state of the implant combining with bone were acquired through 3D reconstructions of micro‐CT. The 3D images, single layer radiographic images, and representative tomographic views were shown in Figure 7B. After implanting for 45 days, both the surface and interior of the HW and HW+DFO scaffolds exhibited a layer of osseous tissue (red). In contrast, only minimal new bone formation was observed in the BCP group, along with the evident fragmentation of the implant. With prolonging the implantation duration to 3 months, the new bone had matured into a continuous structure, and the scaffold had integrated with the host bone in HW and HW+DFO. For the BCP group, there were significant gaps in the defect. Quantitative analysis further supported these findings, and the HW group significantly increased the bone volume fraction (BV/TV) after implanting for 45 days and 3 months compared to the BCP group (Figure 7D,E). Furthermore, the angiogenic capacity of each group was visualized using micro‐CT (Figure 7C). The results of angiography revealed that the bone vessel volume/total volume (BVV/TV) in the HW group was significantly higher than that in the BCP group (Figure 7F). The BV/TV and BVV/TV followed the order HW+DFO > HW > BCP at each time point, indicating the excellent repair ability of HW+DFO.

The mechanical restoration of femoral defects was evaluated by a three‐point bending test after 3 months of implantation. As shown in Figure 7G,H, the HW group could withstand the significantly higher bending load of 82.28 ± 4.78 N than the BCP group (43.23 ± 12.78 N). Moreover, the bending stress of HW (2.17 ± 0.54 MPa) was also significantly higher than that of BCP (1.53 ± 0.45 MPa). The HW+DFO group could withstand the highest bending load (124.27 ± 13.31 N) and the bending stress (4.39 ± 0.47 MPa) among all groups, which reached 40% of the normal femur (334.99 ± 38.95 N). Furthermore, nanoindentation testing was employed to evaluate the mechanical properties of new bone and remaining material. Form the typical loading‐unloading curves of nanoindentations (Figure 7I; Figure S9), the displacements of the HW group were shorter than that in the BCP group, indicating the hardness of the newly formed bone was increased. In addition, the displacements of the newly formed bone in HW+DFO were close to that of control femur. The quantitative statistics revealed that the hardness of new bone (*H*_b_) in the HW group was slightly higher than that in the BCP group (Figure 7J). After implanting for 3 months, the *H*_b_ in the HW+DFO group reached 50% of that of the autologous bone, indicating the excellent ability of type‐H vessel to induce bone regeneration. Furthermore, the hardness and elastic modulus of HW and HW+DFO bioceramics were significantly greater than that of BCP bioceramic (Figure S10).

**FIGURE 8:**
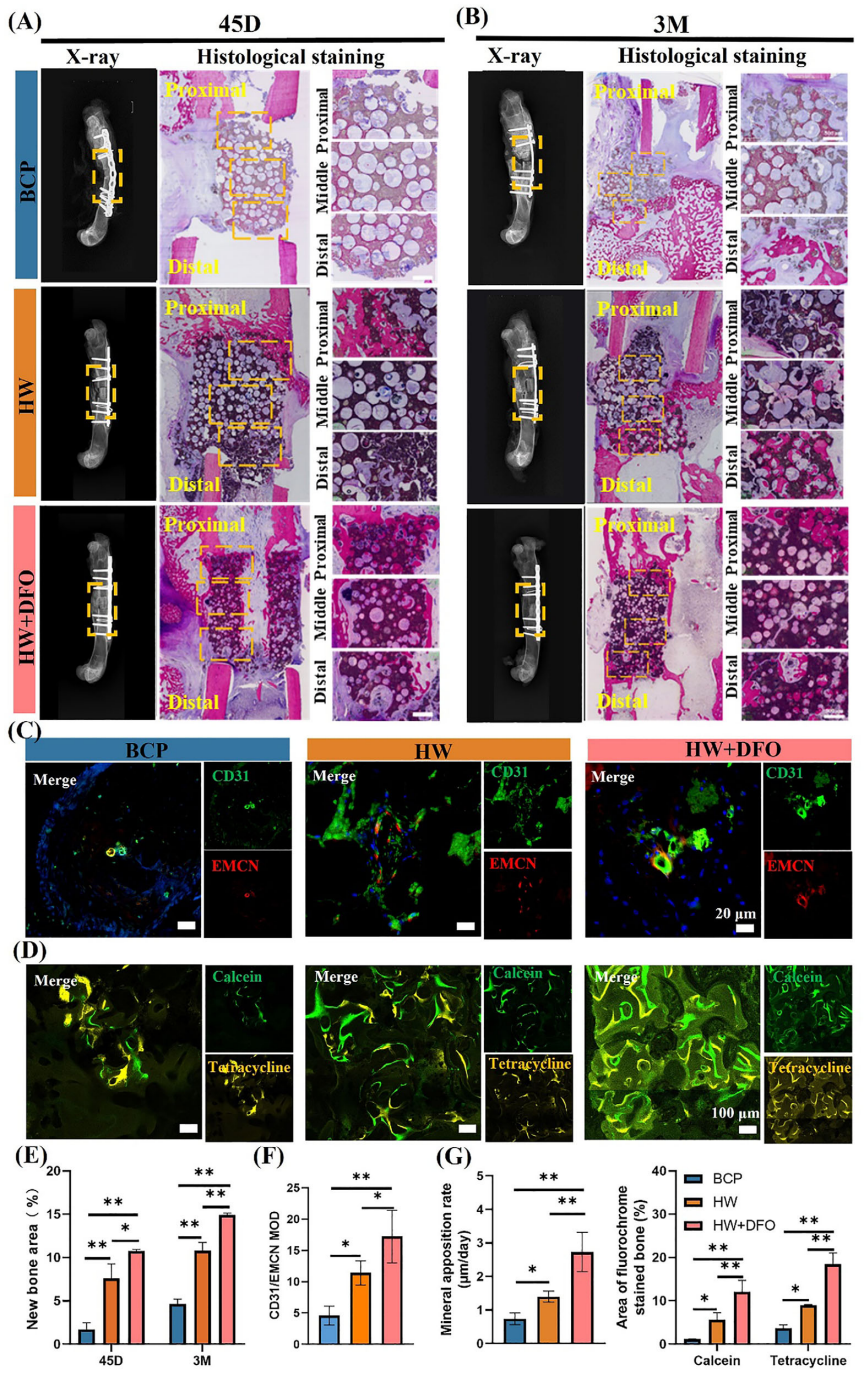
X‐ray images and histological staining (non‐decalcified sections stained with methylene blue and basic magenta) of the defected bone integrated with BCP, HW, and HW+DFO after implanting for (A) 45 days and (B) 3 months (n = 5). (C) Immunofluorescence staining of CD31 and EMCN after implanting for 45 days. (D) Images of calcian (green)—tetracycline (yellow) double fluorescein labeling newly formed bone after implanting for 3 months. Quantitative comparisons of new bone area (E), MOD values (F), and mineral apposition rate and fluorochrome‐labeled new bone area (G) (n = 3). Values are expressed as the mean ± SD. One‐way ANOVA with Tukey's post–hoc test was used for multiple comparisons in (E), (F), and (G). ^*^ *p* < 0.05 and ^**^ *p* < 0.01.

Radiographic evaluation revealed distinct bone healing outcomes among the groups after implantation for 45 days and 3 months. For the BCP group, significant bending of both the fixed steel plate and the force line of femur was observed. In contrast, the HW group maintained stable the fixed steel plate and the force line of femur (Figure 8A,B). The callus formation increased time‐dependently in all groups, and the HW+DFO group had the most relatively complete callus growth at the segmental site. Moreover, the methylene blue and basic magenta staining was performed to evaluate the formation of new bone within the scaffolds. As illustrated in Figure 8A, the BCP group exhibited no obvious new bone tissue at the boundary between the scaffold material and the host bone at 45 days postoperatively, while evident new bone was observed in the HW group. With prolonging the implantation duration to 3 months, BCP group exhibited limited new bone formation, accompanied by material fragmentation. In contrast, abundant newly formed bone was observed in the inner pores of HW, demonstrating the good host bone integration and osteoconductivity (Figure 8B). There was also abundant newly formed bone at the boundary between the scaffold as well as within the scaffold pores in the HW+DFO group. Quantitative statistics showed that the new bone area of HW group was significantly higher than that of the BCP group at each time point, indicating the excellent repair ability of HW (Figure 8E). The quantitative statistics revealed that the new bone area followed the order HW+DFO > HW > BCP at each time point after implanting for 45 days and 3 months. Moreover, SEM images and EDS analysis of the unstained bone section confirmed the effective osseointegration of the HW+DFO with the host bone (Figure S11).

Immunofluorescence staining was carried out to detect the expressions of the type‐H vessels factor of CD31 and EMCN. As shown in Figure 8C, both BCP and HW exhibited the positive expression of CD31 and EMCN after implanting for 45 days. It should be noted that HW could have a significantly higher level of CD31 and EMCN than BCP, indicating HW could promote the type‐H vessels formation inside the implant (Figure 8F). HW+DFO had the highest level of EMCN among the three groups. These results indicated that bamboo‐like whiskers and DFO could synergistically promote the type‐H vessels formation. Furthermore, calcian (green) and tetracycline (yellow) were used to assess the mineralization and new bone formation (Figure 8D). As exhibited in Figure 8G, the area of calcian labeling in HW (5.61 ± 1.64%) was significantly higher than BCP (1.13 ± 0.04%). Moreover, similar phenomena were also observed from tetracycline labeling. Quantitative analysis of the fluorescent marker intervals revealed that the mineral apposition rate in HW was also significantly higher than BCP, which should be responsible to the large new bone area in HW comparing with BCP. HW+DFO exhibited the highest mineral apposition rate, the highest bone formation rate (BFR), and mineralizing surface (MS/BS) among all groups (Figure 8G; Figure S12).

## 3 Discussion

Enhancing the mechanical strength and osteoinduction of Ca‐P ceramics to meet the demands of segmental bone defects has become a research hotspot. Generally, the initial mechanical strength of osteoinductive Ca‐P ceramics fail to meet the demand of segmental bone defects, which poses a significant challenge to the stability of orthopedic implants. This inadequacy, combined with an impaired blood supply, disrupts the balance of osteogenic homeostasis at the defect sites [31].

Inspired by bamboo, the present study proposed a novel strategy to construct in situ bamboo‐like whiskers to achieve the simultaneous enhancements of mechanical properties and bioactivity of Ca‐P bioceramics. After hydrothermal treatment, the surface and interior of BCP bioceramics were entirely replaced by in situ bamboo‐like whisker structures, constructing a unique micro‐nano topography (Figure 1A). The formation of bamboo‐like whiskers could endow HW with high microporosity and specific surface area, which may facilitate the adsorption of relevant functional proteins, thereby activating numerous critical biological processes such as cell adhesion and differentiation [32]. Moreover, the bamboo‐like whiskers significantly enhanced the mechanical properties including the compressive strength and flexural strength of HW ceramics (Figure 1D). The finite element simulation results indicated that bamboo‐like whiskers may effectively mitigate the stress concentration in the ceramic, and significantly alleviate the negative impact of cracks on the mechanical properties (Figure 1G). SEM images revealed that in situ bamboo‐like whiskers could promote crack deflection and enhance toughness (Figure 1H). The mechanism of bamboo‐like whisker‐reinforced may be as follows: 1) when cracks form and propagate in the ceramic matrix, a large number of bamboo‐like whiskers spanning across the crack tips fracture and consume a significant amount of energy, thereby preventing the rapid propagation of the cracks; 2) whisker bridging occurred when whiskers at the crack tip remained intact, impeding further crack propagation and consuming additional energy; 3) as cracks were unable to pass through the whiskers, it became difficult to continue propagating in the original direction and they had to bypass the whiskers, causing crack deflection. The increased crack propagation path led to more energy consumption, which may achieve the effect of mechanical strengthening [33, 34, 35]. Therefore, the presence of in situ bamboo‐like whiskers could redistribute stress at crack tips and increase the energy required for crack propagation via whisker broken, whisker bridging, and crack deflection, thereby enhancing both the strength and toughness of Ca‐P ceramics (Figure 1I). We speculated that the increased fracture energy may be the primary mechanism for the reinforcement of bamboo‐like whiskers. In addition, Zhang et al. have constructed mechanically reinforced HA ceramics by regulating the in situ oriented growth of HA grains using calcium sulfate, and the presence of in situ whiskers significantly enhances the mechanical properties and bioactivity of the HA ceramics to repair supercritical bone defects [36]. Similarly, HW ceramic with a certain initial mechanical strength maintained its structural integrity during the repair of large bone defects. In contrast, BCP ceramic exhibited material fragmentation (Figure 8A,B). These results highlighted that adequate initial mechanical strength was a critical factor for Ca‐P ceramics in the repair of segmental bone defects. The conclusion can be drawn that the initial stability is quite important for Ca‐P ceramics employed in the segmental bone defect, it is expected to expand the application of Ca‐P ceramics in clinical segmental bones in combination with a stable fixation system.

Furthermore, co‐culture model was used to validate the crosstalk of BMSCs and EPCs in vitro. HW+EPC could significantly promote the migration and osteogenic differentiation of BMSCs compared to BCP+EPC (Figure 3). This confirmed that HW‐mediated differentiation of EPCs could secret several growth factors such as Noggin, thereby enhancing the osteogenic differentiation of BMSCs. More intriguingly, compared to BCP+BMSC, HW+BMSC also could significantly facilitate the tube formation and migration of EPCs. This indicated that HW‐mediated osteogenic differentiation of BMSCs could lead to the secretion of VEGF that promote angiogenesis (Figure 4). These findings were consistent with the superior in vivo osteogenic performance and increased formation of type‐H vessels observed with HW (Figures 6 and 8). Therefore, bamboo‐like whiskers could enhance the communication between EPCs and BMSCs, and amplify the ability of EPCs to recruit BMSCs, thereby enhancing the osteoinductivity and type‐H vessel formation of HW.

Another challenge in repairing large bone defects stems from the complex interplay between bone regeneration and vascularization [37]. The type‐H vessel confirmed the direct molecular interactions between ECs and osteoblastic cells. On one hand, type‐H ECs in mice with activated Notch signaling exhibit increased secretion of Noggin, which plays a positive role in initiating the cartilage‐to‐bone transformation. On the other hand, osteoblasts surrounding type‐H vessels also secrete VEGF, thereby promoting angiogenesis [24, 25]. In the present study, immunofluorescence staining and qRT‐PCR confirmed that in situ bamboo‐like whiskers could promote the differentiation of EPCs into type‐H ECs (Figure 2E–G). To investigate the molecular mechanisms by which in situ bamboo‐like whiskers regulate EPCs, RNA sequencing results indicated that HW could upregulate the expression of integrin‐related genes in EPCs and promote the formation of type‐H ECs via the Hif‐1α signaling pathway (Figure 5). To the best of our knowledge, integrin signaling was closely associated with material surface topography, which could regulate cellular biological behaviors [38, 39]. The HIF‐1α signaling pathway could respond to hypoxia and play a crucial role in regulating neovascularization [40]. Moreover, Groen et al. revealed that HIF‐1α signaling and integrin cell surface interactions were identified as major signaling pathways in the molecular control of tissue regeneration for Ca‐P materials [41]. From the in vivo experiments in Figure 6E, there was more Integrin b1 expression in HW than BCP, further certifying the results of RNA sequencing. Therefore, the unique bamboo‐like whiskers of HW may influence cell adhesion and spreading upregulate integrin gene expression, thereby activating the downstream HIF‐1α signaling pathway and ultimately promoting the differentiation of EPCs into type‐H ECs.

To further enhance the type‐H vessels formation potential of the HW scaffold, we employed capillary force to efficiently and uniformly capture DFO within in situ bamboo‐like whiskers [42, 43]. Compared to BCP, HW not only enhanced the DFO adsorption but also enabled sustained DFO release through its unique bamboo‐like whiskers (Figure 1J–L). HW+DFO induced extensive vascular formation not only at the defect periphery but also near the defect center, indicating that the initial release of DFO rapidly promoted type‐H vessels ingrowth into the defect region (Figure 7). Numerous type‐H vessels and migrated osteoprogenitor cells or osteoblasts were distributed throughout the bioceramics, while in situ bamboo‐like whiskers further enhanced new bone formation. Furthermore, histological evaluation and micro‐CT analysis conclusively demonstrated that HW+DFO could exhibit significantly higher osteogenic capability compared to HW and BCP, indicating its immense potential for repairing segmental bone defects (Figure 8). Yan et al. [44] have constructed vascularized 3D printed scaffolds that could enhance vascular and bone regeneration in a rat femoral defect model by releasing DFO to increase HIF‐1α levels. Cui et al. [45, 46] also reported that the construction of 3D biomimetic scaffolds could not only promote type‐H vessels formation but also regulate the biological behaviors of osteoclasts and BMSCs, thereby enhancing the regeneration of bone defects. Although DFO may affect other cells, such as macrophage and osteoclast, the findings emphasized the crucial roles of HIF‐1α in modulating type‐H vessels formation. These findings suggested that rapid induction of type‐H vessel formation to establish an optimal osteogenic microenvironment was crucial for large segmental bone defect regeneration. The dynamic changes of type‐H vessels may be closely related to the regeneration of new bone tissue in the early stage, and could guide the direction of new bone maturation. Type‐H vessels could be remodeling into mature vessels as extension of time. In addition, mechanical evaluation of the implanted ceramics revealed that HW+DFO could exhibit significantly higher flexural strength and new bone hardness compared to BCP and HW (Figure 7G–J). After implantation, the inorganic ceramic material gradually transformed into an organic/inorganic composite through continuous ingrowth of fibrous connective tissue and new bone [47, 48]. HW+DFO could promote rapid formation of type‐H vessel, which secreted osteogenic factors and physically interacted with osteoprogenitors, thereby facilitating new bone formation. Comparing to the present nonbiodegradable metallic implants, HW+DFO were biodegradable, which could be replaced by the new bone gradually. A certain degree of degradation at the junction between the material and the host bone was observed in the previous study at 3 months. Future research will focus on the degradation rate of the materials implanted for a long period of time. Consequently, HW+DFO demonstrated superior bone regeneration capacity in the repair of large segmental bone defects, with newly formed bone tissue fully occupying the entire scaffold within just three months. Overall, bamboo‐inspired whisker‐reinforced bioceramics could promote rapid type‐H vessels formation and achieve excellent repair in the segmental bone defects.

## 4 Conclusion

Inspired by bamboo, the present study successfully developed bamboo‐like whiskers‐reinforced bioceramics to accelerate large segmental bone regeneration. The study investigated the mechanisms of mechanical enhancement effects through in situ bamboo‐like whiskers. It was found that the in situ bamboo‐like whiskers effectively reduced stress concentration within the porous ceramics, and inhibited crack propagation within the ceramic matrix via whisker broken, whisker bridging, and crack deflection, resulting in 3 times increase in the compressive strength. In addition, HW could facilitate the type‐H ECs formation by regulating the integrin/Hif‐1α signaling cascade pathway. Type‐H ECs could promote the osteogenic differentiation of BMSCs through coupling factors, and create a suitable repair environment for osteogenesis. Moreover, bamboo‐like whiskers also served as a localized delivery platform for DFO to further regulate the rapid formation of type‐H vessels. In vivo experiments confirmed that HW+DFO could regulate type‐H vessels and osteoinduction to promote large segmental bone regeneration with excellent biomechanical recovery. The innovative approach combined type‐H vessels modulation and osteoinduction enhancement, offering a promising solution for the clinical repair of segmental bone defects.

## 5 Material and Methods

### 5.1 Material Preparation and Characterization

BCP porous ceramics (BCP) with HA/β‐TCP phase ratio of 20/80 were fabricated as reported in our previous work [49]. In brief, BCP powder was synthesized by a chemical precipitation method in National Engineering Research Center for Biomaterials (Sichuan University, China). BCP porous ceramics were prepared using the hydrogen peroxide (H_2_O_2_) foaming method and sintered at 1100°C for 2 h. Then, BCP ceramics underwent the specifically hydrothermal treatments to construct the in situ bamboo‐like whiskers. The as‐prepared BCP ceramics were immersed in 0.2 mol L^−1^ trisodium phosphate (Na_3_PO_4_) and heated at 180°C for 24 h into the Teflon autoclaves. After hydrothermal treatment, the obtained ceramics were washed with deionized water and dried at 60°C, denoted as HW. Bioceramics were cut into circular disks (Φ 8 × 2 mm) for DFO adsorption and cell culture, cylinders (Φ 2 × 3 mm) for intramuscular implantation in mice, and cylinders (Φ 6 × 10 mm) for femoral segmental bone implantation in rabbit. The surface morphologies of the obtained bioceramics were observed by a scanning electron microscopy (SEM, JSE‐5900LV, Japan). 3D Optical Profiler (Sneox 090, Spanish) was employed to evaluate the surface roughness of samples. The specific surface area (SSA) was characterized by a surface area analyzer (Gemini VII 2390t, USA). X‐ray diffraction (XRD, Empyrean PANalytical) with CuKα raidiation at a current of 20 mA and voltage of 30 kV was used to determine the phase composition of the sample. And the porosity and pore size distribution of ceramics were characterized by a mercury porosimetry (AutoPore IV 9500, Micromeritics). Moreover, the mechanical strengths of the obtained bioceramics were measured by a universal testing machine (Instron 5967, America) with the loading rate of 0.5 mm min^−1^. Bioceramics were cut into cylinders (Φ 5 × 7.5 mm) for compressive mechanical tests, and bulks (3 × 4 × 30 mm) for three‐point bending tests. Each group included five parallel specimens. Zeta potential of BCP and HW was assessed using an electrokinetic analyzer (SurpassTM 3, Anton‐Paar, Austria), a device capable of measuring zeta potential of the materials.

### 5.2 Ca^2+^ Release Measurement

For Ca^2+^ release kinetic analysis, the ceramics were immersed in α‐minimum essential medium (α‐MEM, Servicebio, China) at the ratio of 100 mg mL^−1^. The procedure was executed at 37°C within a thermostatic oscillator. At each determining point, a certain amount of submersed mixture was removed to measure the Ca^2+^ concentration via a Calcium Assay Kit (Njjcbio, China). It was replaced with an equivalent volume of fresh α‐MEM solution.

### 5.3 DFO Adsorption and Release

BCP or HW were immersed in a 1 mg mL^−1^ DFO solution and incubated at 37°C for 2 h. HWwith DFO adsorption were denoted as HW+DFO. The adsorption amount of DFO on the ceramics was calculated by using an ultraviolet spectrophotometer (UV‐3600, Shimadzu). For DFO release kinetics analysis, the samples with DFO adsorbing were transferred into deionized water, and placed on a constant temperature shaker (37°C, 100 Hz). All the immersed solution were collected at each measurement point to measure the concentration of DFO. It was simultaneously replaced with an equal volume of fresh deionized water. Each experiment was performed in triplicate, with each sample measured three times.

### 5.4 Finite Element Analysis

The mechanical simulation of obtained bioceramics was evaluated using COMSOL Multiphysics 5.6 software based on our previous research [50]. Considering the model as an isotropic elastic material, the elastic modulus is uniformly set to 51.5 GPa, and the Poisson's ratio to 0.28. A simplified 2D monolayer model was adopted to facilitate the analysis. In this model, the in situ bamboo‐like whiskers were simplified as columnar shapes. A compressive stress of 1 MPa was applied to the surface.

### 5.5 In‐Vitro Cellular Culturing

BCP ceramic disks (Φ8 × 2 mm) were seeded by rat endothelial progenitor cells (EPCs, iCell Bioscience, China) in 48‐well plates with EGM‐2 media supplemented with growth factor bullet kit (Lonza, Germany). Moreover, mouse bone marrow mesenchymal stem cells (BMSCs, Cyagen Biosciences, China) were cultured with α‐MEM containing 10% fetal bovine serum (FBS, Gibco, USA) and 1% penicillin/streptomycin (PS, Gibco, USA). In the co‐culture system, BCP or HW and EPCs were directly co‐cultured on the upper chamber of the Transwell system with a 0.4 µm pore‐size (Corning, USA), and BMSCs were plated on the lower chamber. Moreover, BCP or HW and BMSCs were directly co‐cultured on the upper chamber of the Transwell system, and EPCs were plated on the lower chamber. The protein levels of VEGF and Noggin in conditioned medium were examined with ELISA kits (Jiangsu Meibiao Biotechnology Co., Ltd, China) according to the manufacturer's instructions.

### 5.6 Cell Proliferation and Morphology

The proliferation of EPCs on the bioceramics was quantified by using CCK‐8 method after cultured for 1, 3 and 5 days, and the cell density was 1 × 10^4^ cells per wall. Moreover, the live‐dead staining was characterized by fluorescein diacetate (FDA; Sigma, USA) and propidium iodide (PI; Sigma, USA), and then detected by a laser scanning confocal microscopy (CLSM, Zeiss, Germany). In order to observe the adhesion and spreading of EPCs on the bioceramics, the samples were dehydrated and dried after cultured for 2 days, then characterized by SEM.

### 5.7 Immunofluorescent Staining, Western Blot, and Gene Expression

Immunofluorescent staining of the related type‐H vessels proteins of CD31 and EMCN was carried out after culturing for 7 days according to the previous literature [30]. The immunofluorescent staining was performed using CD31 (Santa, USA), and EMCN (Immunoway, USA) primary antibody and goat anti‐rabbit secondary antibody (HuaAn, China), and goat anti‐mouse secondary antibody (HuaAn, China). The cell nuclei were counterstained with DAPI (Solarbio, China). CLSM was employed to capture the immunofluorescent staining images, and the mean optical density (MOD) was analyzed by Image‐Pro Plus software. Western blot (WB) was performed using Integrin β1 (Abclonal, China) and HIF‐1α (Zenbio, China) antibody. Western Lightning chemiluminescence kit (SuperKine, China) and ChemiDoc XRS+ system (Bio‐Rad, USA) were used to visulalize the protein bands. Real‐time reverse‐transcript PCR (qRT‐PCR) method was employed to measure the type‐H vessels‐related gene expressions of *Cd31*, *Emcn*, *Noggin*, and *Vegf*. The primer sequences were included in Table S1. EPCs were seeded on BCP and HW with a density of 1 × 10^5^ cells per well and cultured for 4, and 7 days. The detailed experiment procedures could be referred to our previous work [51].

### 5.8 Transcriptome Sequencing Analysis

EPCs were cultured on the BCP and HW ceramics for 7 days, and lysed with the Invitrogen TRIzol Reagent for transcriptome analysis. The RNA sequencing was assessed using Illumina NovaSeq 6000 (Illumina, USA). Differentially expressed genes (DEGs) analysis of two groups was performed using the DESeq2 R package. Then, the thresholds were set as *p* value < 0.05 and fold change > 2 for the selection of the significantly DEGs. To further investigate the differentially expressed genes, the Oebiotech Cloud Platform (Shanghai OE Biotech Co., Ltd, China) was used to visualize Kyoto Encyclopedia of Genes and Genomes (KEGG) pathway.

### 5.9 In Vitro EPCs Migration and Tube Formation Assay

To assess in vitro osteogenic‐angiogenic coupling, culture media was used to induce EPCs migration and tube formation. The culture media were collected from BMSCs/BCP, and BMSCs/HW every 2 days, and centrifuged to extract supernatants. For cell migration, EPCs (5 × 10^4^ cells) were seeded into a 48‐well plate and incubated until confluence. The 200 µL sterile micropipette tip was used to make a liner scratch wound across. After removing the de‐attached cells using PBS washing, culture media containing 2% FBS was added into each well and incubated for 12 h. Finally, the images of cell migration were captured by an inverted microscope at 0 h, and 12 h, respectively. For tube formation, 50 µL Matrigel (Coring, USA) was spread evenly in a 96‐well plate, and incubated for 30 min at 37°C. Then, EPCs (4 × 10^4^ cells) were implanted in each well, and treated with different culture media. Pictures were obtained using an inverted microscope after 6 h treatment. The scratch area and tubule formation tube data were measured by ImageJ software. Each group has three parallel samples.

### 5.10 Trans‐Well Migration Assay of BMSCs

The culture media were used to investigate the effects on the migration of BMSCs. BMSCs were seeded at a density of 2 × 10^4^ in the upper chamber in a serum‐free medium. The culture media were collected from EPCs/BCP, and EPCs/HW every 2 days, and centrifuged to extract supernatants. Then, 500 µL of culture media were added to the lower chamber. After 24 h of co‐culture, the cells were fixed with 4% paraformaldehyde and stained with 0.1% crystal violet. The cells were observed and photographed using an optical microscope.

### 5.11 In Vitro Osteogenic Differentiation of BMSCs

To assess in vitro osteogenic‐angiogenic coupling, BMSCs were seeded on the lower chamber, and BCP/ EPCs or HW/ EPCs were seeded on the upper chamber in the co‐culture system. Alkaline phosphatase (ALP) staining kit (Beyotime, China) was utilized for ALP staining after 4 days. Immunofluorescent staining of the osteogenic specific proteins, such as bone morphogenetic protein 2 (BMP2) and osteocalcin (OCN), was carried out after co‐culture system for 7 days. The samples were treated by using BMP2 (Abcam, UK), and OCN (Abcam, UK) primary antibody and goat anti‐rabbit secondary antibody (Proteintech, USA). CLSM was finally employed to capture the Digital images. Moreover, qRT‐PCR was employed to evaluate measure the gene expression related to osteogenesis, including runt‐related transcription factor 2 (*Runx2*), collagen I (*Col‐1*), *Bmp2*, osteocalcine (*Ocn*), and osteopontin (*Opn*). The primer sequences were included in Table S2.

### 5.12 Mice Intramuscular Implantation

The muscle model for mice was used to evaluate the osteoinduction of the obtained bioceramics, which was approved by the Institutional Animal Care and Use Committee (IACUC) of Sichuan University (NO. KS2024001). The female BALB/C mice (four weeks old, weight 20 g) were purchased from Sichuan Province Medical Experimental Animal Center of China. As previously described, BCP or HW bioceramics were implanted into the muscular pouches which were created by blunt dissection after mice anesthetization (n = 6) [52]. At 2, 4 weeks, and 3 months postoperatively, the mice were sacrificed, and the samples were harvested for further analysis. In the ultrasound images, the varying brightness of materials and tissues could determine their location.

### 5.13 Rabbit Femoral Segmental Bone Implantation

This animal experiment was approved and guided by the IACUC of Sichuan University (No. KS2024001). Thirty‐six adult female New Zealand white rabbits (3–4 months old, about 2.5 kg) were randomly divided into the BCP, HW, and HW+DFO group (n = 6 per group). The surgical process was similarly reported in our previous work [53]. Briefly, after general anesthesia with sodium pentobarbital (20 mg kg^−1^ body weight), the surgical area was prepared to expose the femur. A 10 mm long segmental defect was created in the middle of the femur, and the material was tightly placed into the created segmental bone defect. The implant was fixed using a unilateral stainless‐steel plate and screws. After 45 days and 3 months of implantation, the samples were harvested for radiographic, histological staining, and biomechanical analysis.

### 5.14 Histological and Immunofluorescent Analysis

All the harvested explants were fixed in 4% paraformaldehyde. For soft‐tissue slices, the samples were then decalcified in 10% EDTA. After dehydration, embedding, and sectioning, the sections were stained with H&E staining for histomorphological analysis. Besides, the protein expressions of CD31 (Abcam, UK) and EMCN (Biorbyt, UK) were also performed by immunofluorescence staining [54]. For non‐decalcified histology, the specimens from the rabbit femoral segmental bone implantation were stained by methylene blue and basic fuchsin. Finally, all staining images were scanned by a light scanner (VS200, Olympus, Japan), and analyzed by IPP 6.0 software.

### 5.15 Micro‐CT Analysis

At 45 days and 3 months post‐surgery, the rabbits were humanely euthanized and the femurs were harvested. After fixation in 4% paraformaldehyde, the specimens were examined using the digital X‐ray system (Canon, Japan). Additionally, after removal of the plate and screw, Micro‐CT (Scanco Medical AG, Switzerland) was utilized to evaluate new bone growth at the defect site. The 3D reconstructions were reconstructed using the Materialise Mimics and Imaris software for quantitative analysis.

### 5.16 Microvascular Angiography

Microfil contrast agent (FlowTech, USA) was used for microvascular angiography to investigate the vascular regeneration induced by the bioceramics in the defects, which was described previously. Micro‐CT was performed to detect the vascularization after decalcification, and the blood vessels were distinguished by red color based on different threshold values. The 3D reconstructions were analyzed using Imaris 10.1 software to quantify vessel volume fraction (BVV/TV).

### 5.17 Sequential Fluorescent Labeling

To assess the bone formation and mineralization process, sequential labeling with two calcium‐binding fluorochromes was utilized. At 8 and 10 weeks after surgery, tetracycline (Solarbio, 50 mg mL^−1^, 50 mg kg^−1^) and calcein green (Solarbio, 10 mg mL^−1^, 10 mg kg^−1^) were used to label the new bone via intraperitoneal injection. At 12 weeks after surgery, the samples were harvested for hard tissue sectioning, and observed by CLSM.

### 5.18 Biomechanical Tests

A universal mechanics tester (Instron 5967, America) was performed to detect the three‐point bending testing of the specimens after the plate and screw were removed. The test parameters were configured as follows: loading rate of 0.5 mm min^−1^, and test distance of 3.0 cm. Stress–strain curves were recorded, and the maximum bending load and bending stress were calculated. In addition, nanoindentation tests were carried out on the femur specimens using a G200 nanoindenter system (Agilent Technologies Inc., Chandler, AZ). Briefly, a Berkovich diamond tip was used to indent the surface of the samples with a maximum load of 10 mN and a loading/unloading rate of 20 mN min^−1^. Continuous stiffness measurements were performed under the guidance of an optical microscope to determine the elastic modulus (*E*) and hardness (*H*) of the newly formed bone and the remaining materials within the defect region.

### 5.19 Statistical Analysis

Each quantitative result was derived from at least triplicate measurements (n ≥ 3), and presented as the mean ± standard deviation (SD). No data preprocessing was performed. The sample size (n) for each statistical analysis was reported in the corresponding “figure legends”. The statistical analysis was conducted based on GraphPad Prism 8.0 (GraphPad Software Inc, La Jolla, CA, USA). Comparisons between two groups were performed using the Student's *t*‐test (two‐sided), while comparisons among multiple groups were analyzed using one‐way analysis of variance (ANOVA) with Tukey's multiple comparison test. Statistical significance was set at *p* < 0.05. The label ^*^ was utilized to represent statistical significance.

## Funding

This work was sponsored by Advanced Materials‐National Science and Technology Major Project of China (Grant Nos. 2025ZD0619000, 2025ZD0619003), Sichuan Province Science and Technology Project (Grant Nos. 2022ZDZX0029, 2025ZDZX0128, and 2025YFMS0020), and National Science Foundation of China (Grant No. 52372269).

## Conflicts of Interest

The authors declare no conflicts of interest.

## Supporting information




**Supporting File**: advs75178‐sup‐0001‐SuppMat.docx.

## Data Availability

The data that support the findings of this study are available in the supplementary material of this article.
