# Supplementary material for: Bamboo‐Like Whiskers‐Reinforced Bioceramics Accelerate Large Segmental Bone Regeneration via Dual Modulation of Type‐H Vessels and Osteoinduction
Source: Adv Sci (Weinh). 2026 Apr 7:e75178. Online ahead of print. doi: 10.1002/advs.75178 (PMC13334676; doi:10.1002/advs.75178)
Supplement: Supplementary file 1 — Supporting File: advs75178‐sup‐0001‐SuppMat.docx. [file ADVS-9999-e75178-s001.docx]

Supporting Information

Bamboo-like whiskers-reinforced bioceramics accelerate large segmental bone regeneration *via* dual modulation of type-H vessels and osteoinduction

Cong Feng, Haibo Teng, Chuyao Xu, Jiaze He, Keting Liu, Xueying Li, Kai Zhang, Xiangdong Zhu^*^, Xiangfeng Li^*^, Jianguo Xu, Xingdong Zhang

**Figure S1** SEM images of cross-sections for HW.

*
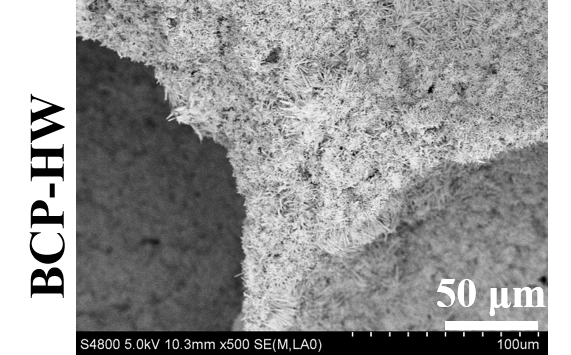
*

**Figure S2** Concentrations of Ca^2+^ in α-MEM solution released from BCP and HW.

**
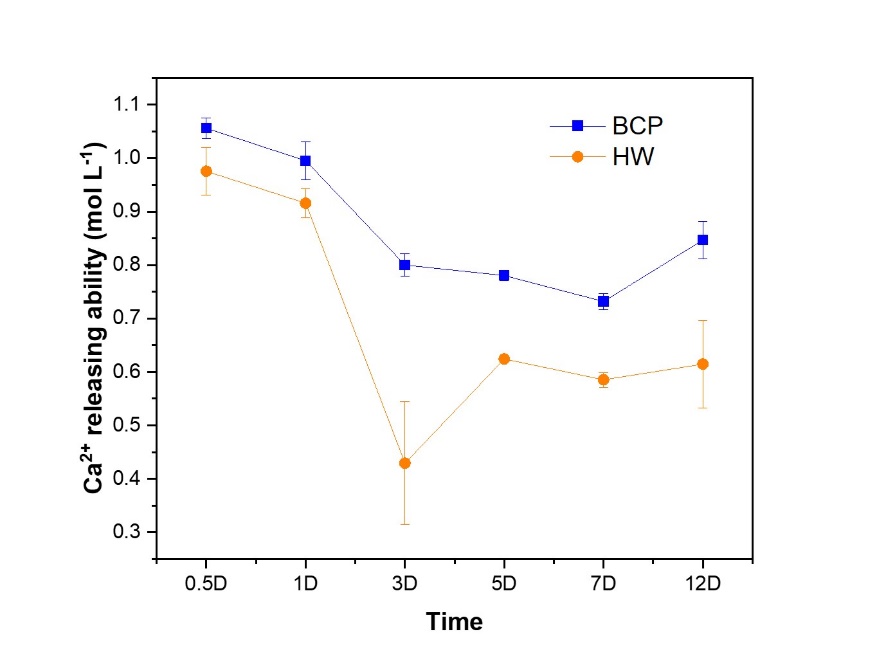
**

**Figure S3** Compressive modulus of BCP and HW (n = 3). Values are expressed as the mean ± SD; **p* < 0.05, ***p* < 0.01.


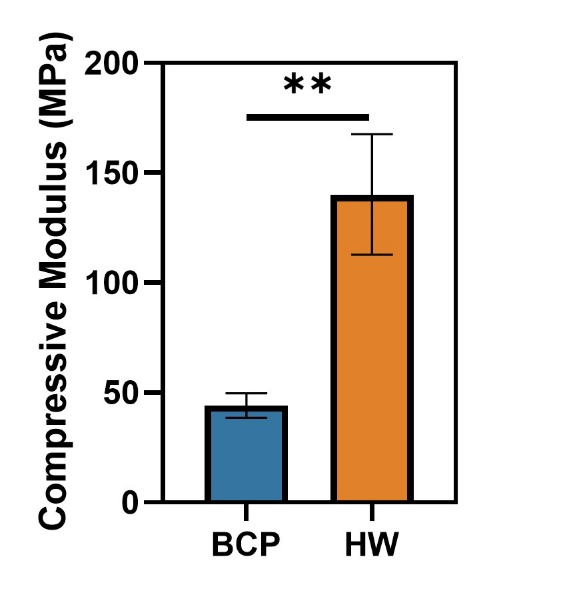


**Figure S4** ALP staining of BMSCs cultured with different conditioned medium and extract.


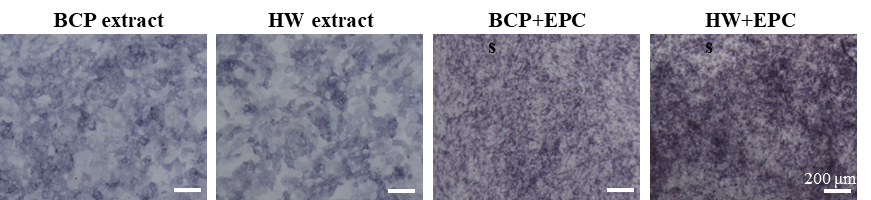


The conditioned medium could significantly increase ALP activity compared to the extract, suggesting the osteogenic differentiation of BMSCs was dependent on the formation of type H ECs. HW conditioned medium could increase ALP activity compared to BCP conditioned medium.

**Figure S5** ELISA results of the key paracrine factors (n = 5). Values are expressed as the mean ± SD; **p* < 0.05, ***p* < 0.01.


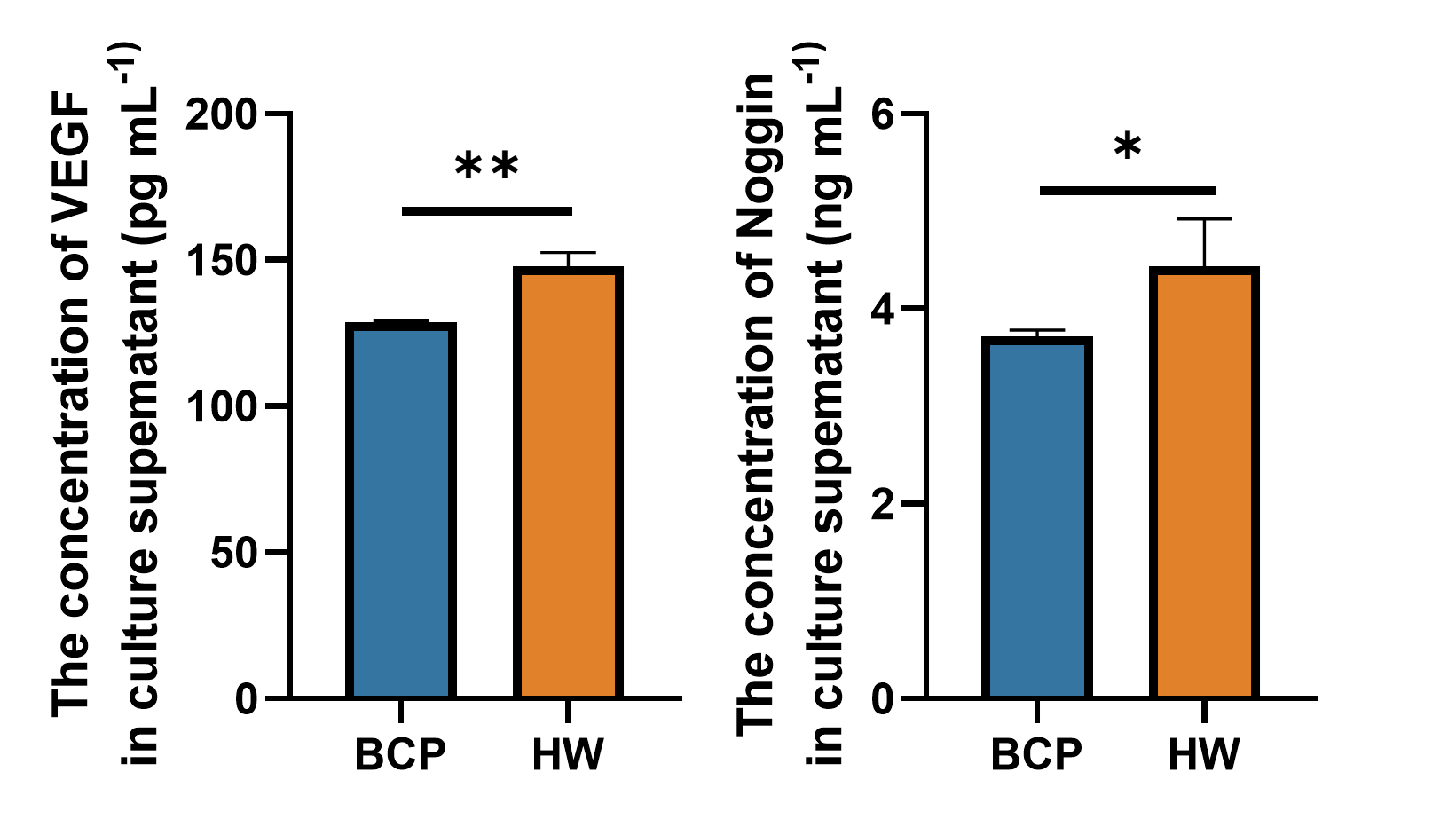


**Figure S6** qRT-PCR analysis for integrin binding gene expressions of EPCs from different groups on day 4 (n = 3). Values are expressed as the mean ± SD; **p* < 0.05, ***p* < 0.01.

qRT-PCR results revealed that HW group could significantly expressed higher expression of *Intgαv* compared to the BCP group.

**Figure S7** Immunofluorescence staining of HIF-1α of samples after 2 weeks of implantation.

**
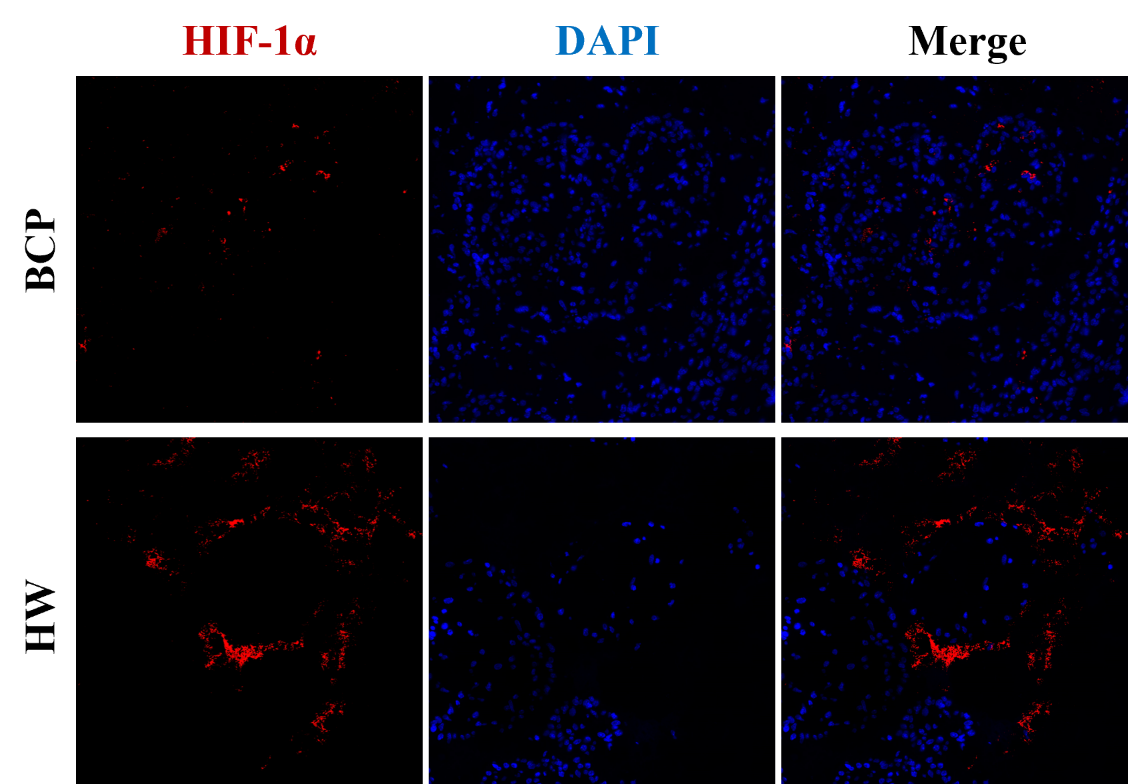
**

**Figure S8** Immunofluorescent staining of EMCN and osterix^+^ osteoprogenitor cells.


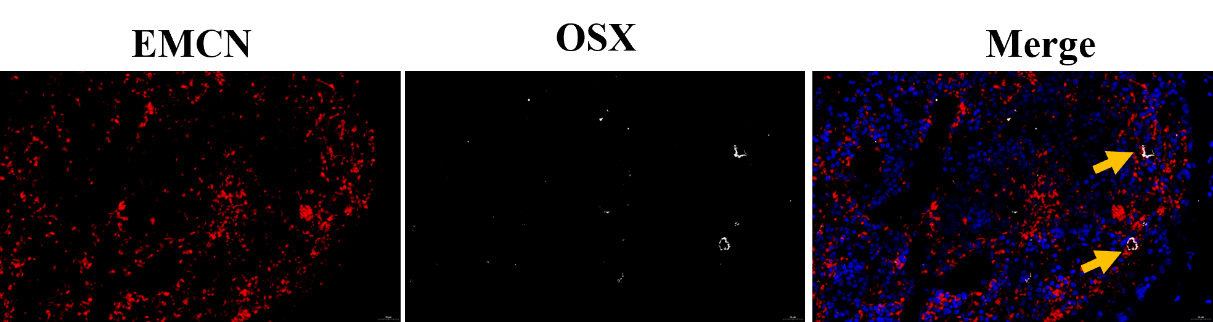


**Figure S9** Typical load–displacement curves generated from nanoindentation tests on remaining material.


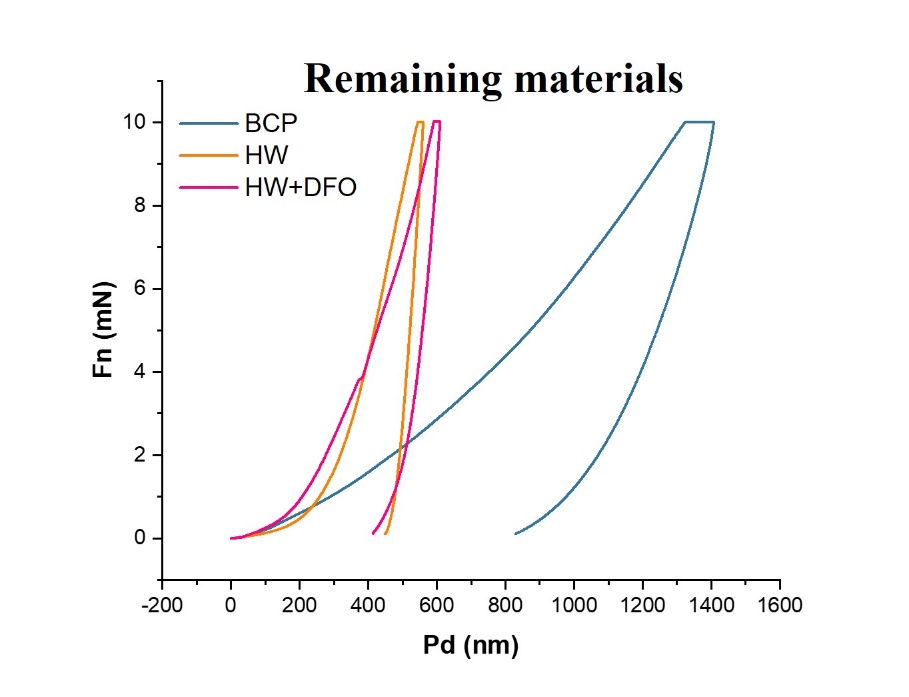


**Figure S10** Quantitative analysis of averaged hardness and elastic modulus of remaining material (n = 3). Values are expressed as the mean ± SD; **p* < 0.05, ***p* < 0.01.


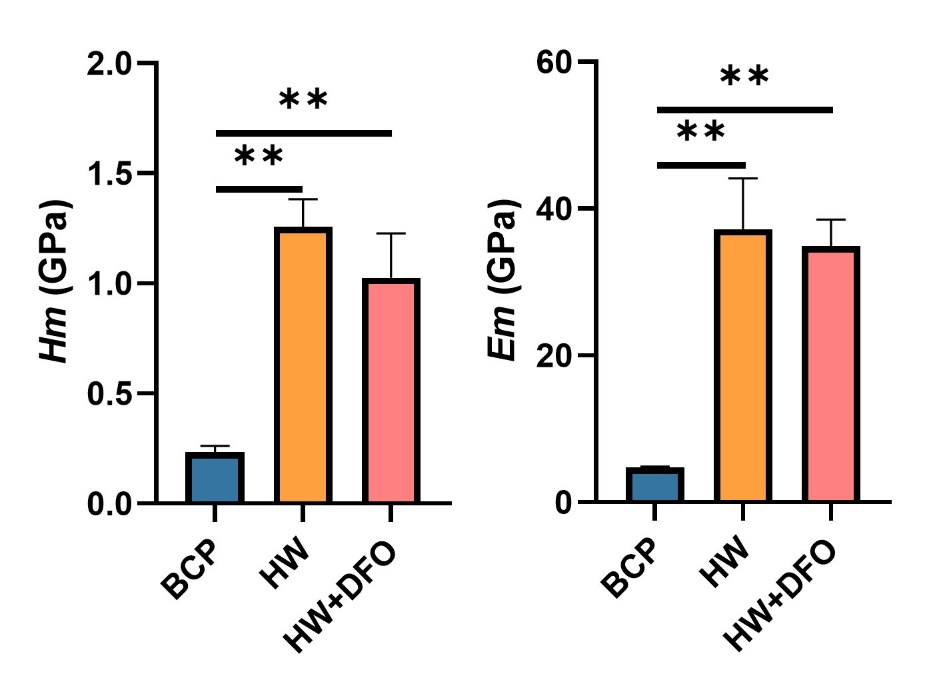


**Figure S11** SEM and EDS observations of the interface between new bone and residual material.


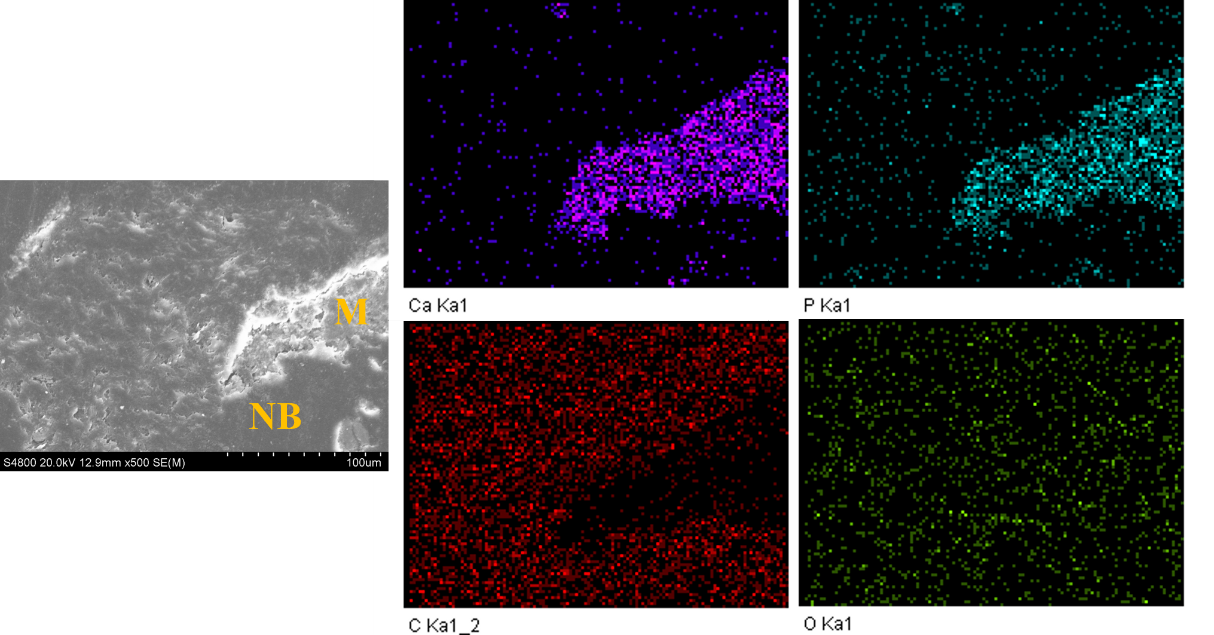


**Figure S12** Quantitative comparisons of bone formation rate (BFR) (n = 3). Values are expressed as the mean ± SD; **p* < 0.05 and ***p* < 0.01.


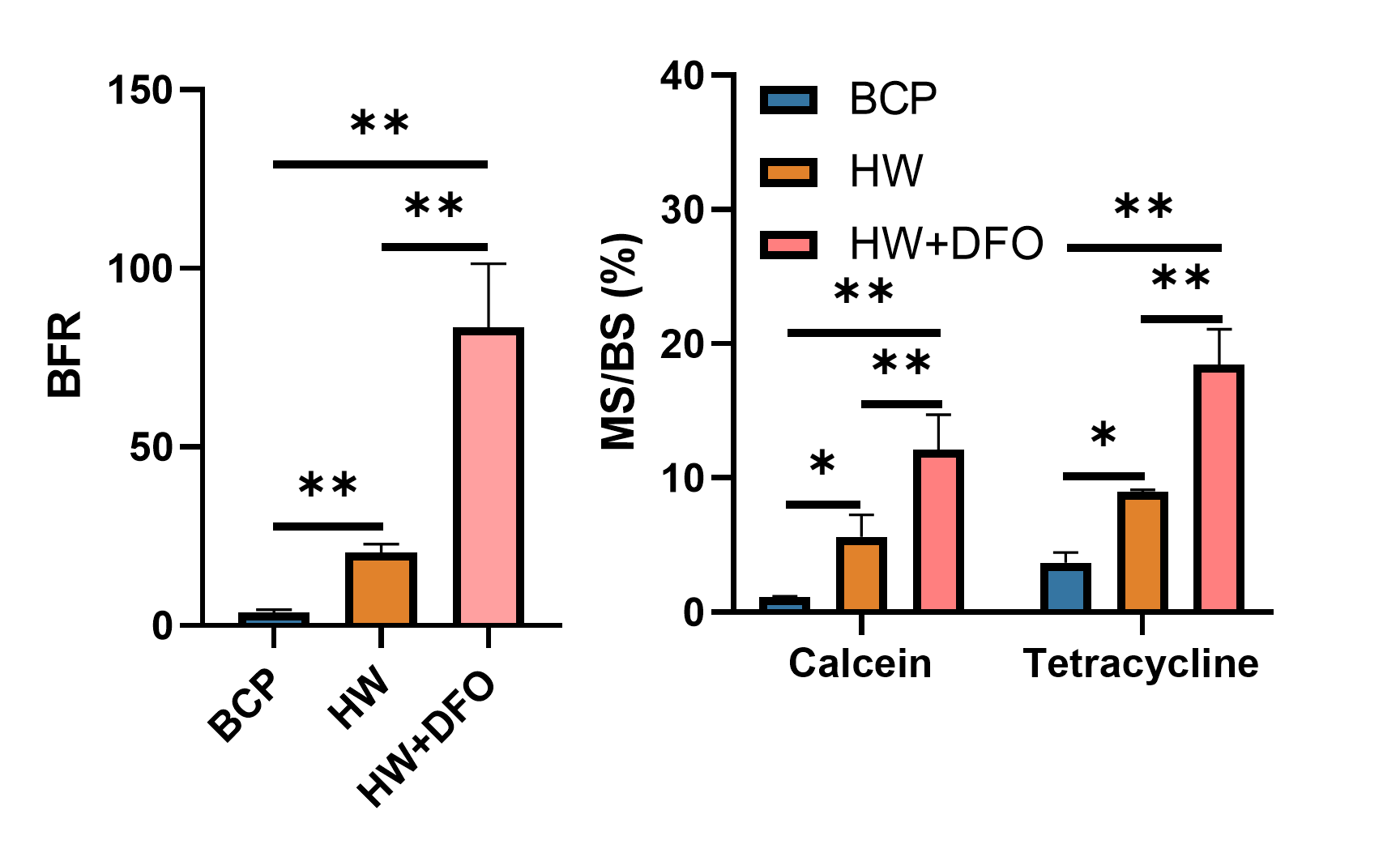


**Table S1** Table of primers utilized for qRT-PCR amplification

| Gene | 5' to 3 | Primers |
| --- | --- | --- |
| *Cd31* | Sense | 5’-AGTAGCATCCTGGTCAACATAACA-3' |
|  | Anti-Sense | 5’-ACAACACCGTCTCTTCCTTCTG-3' |
| *Emcn* | Sense | 5’-TTCACCGACAACACCAAAACA-3' |
|  | Anti-Sense | 5’-AACTCTGATTCTCCGTCTTGTTC-3' |
| *Bfgf* | Sense | 5’-ACTTCGCTTCCCGCACT-3' |
|  | Anti-Sense | 5’-TGTGGGTCGCTCTTCTCC-3' |
| *Pdgf* | Sense | 5’-CGTGACTTTCTGGGAGGA-3' |
|  | Anti-Sense | 5’-CTCTGCCCATCGAGTTCT-3' |
| *Noggin* | Sense | 5’-ATGCCGAGCGAGATCAA-3' |
|  | Anti-Sense | 5’-GTACAGCACCGGGCAGA-3' |
| *Vegf* | Sense | 5’-GAACTTTCTGCTGTCTTGGGTG-3' |
|  | Anti-Sense | 5’-GGATGGCTTGAAGATGTACTCG-3' |
| *Notch* | Sense | 5’-AGGCTCTGCCGACATCA-3' |
|  | Anti-Sense | 5’-AGGAAGGGGTGCTCTGG-3' |
| *Intg**a6* | Sense | 5’-GAACCCGATGAAGGCTGGAA-3' |
|  | Anti-Sense | 5’-TGTAAGACACGACTGGGCTG-3' |
| *Intga1* | Sense | 5’-TTGCCTTGCTGCTGATTTGG-3' |
|  | Anti-Sense | 5’-AGTTGTCACGGCACTCTTGT-3' |
| *Intga5* | Sense | 5'-TGACCAGGTTCTGCAGGATAGA-3' |
|  | Anti-Sense | 5'-AGTAGAAATTGCAGCCACAGAGTAAC-3' |
| *Gapdh* | Sense | 5’-CGGAGTCAACGGATTTGGTCGTAT-3' |
|  | Anti-Sense | 5’-AGCCTTCTCCATGGTGGTGAAGAC-3' |

**Table S2** Table of primers utilized for qRT-PCR amplification

| Gene | 5' to 3 | Primers |
| --- | --- | --- |
| *Bmp2* | Sense | 5’-GAGGAGAAGCCAGGTGTCT-3’ |
|  | Anti-Sense | 5’-GTCCACATACAAAGGGTGC-3’ |
| *Ocn* | Sense | 5'-GGGGGCTGGGGCTCCAAGT-3' |
|  | Anti-Sense | 5'-TGGTGAAGACGCCAGTAGACTC-3' |
| *Col-1* | Sense | 5'- ACAGACGAACAACCCAAACT-3' |
|  | Anti-Sense | 5'- GGTTTTTGGTCACGTTCAGT-3' |
| *Runx-2* | Sense | 5’-AGCCTCTTCAGCGCAGTGAC-3’ |
|  | Anti-Sense | 5’-CTGGTGCTCGGATCCCAAA-3’ |
| *Opn* | Sense | 5'-CCAAGCGTGGAAACACACAGCC-3' |
|  | Anti-Sense | 5'-GGCTTTGGAACTCGCCTGACTG-3' |
| *Gapdh* | Sense | 5'-GGCAAGTTCAACGGCACAGT-3' |
|  | Anti-Sense | 5'-TGGTGAAGACGCCAGTAGACTC-3' |
